# Selection and validation of reference genes for quantitative expression analysis of miRNAs and mRNAs in Poplar

**DOI:** 10.1186/s13007-019-0420-1

**Published:** 2019-04-06

**Authors:** Fang Tang, Liwei Chu, Wenbo Shu, Xuejiao He, Lijuan Wang, Mengzhu Lu

**Affiliations:** 1 State Key Laboratory of Tree Genetics and Breeding, Key Laboratory of Tree Breeding and Cultivation of the National Forestry and Grassland Administration, Research Institute of Forestry, Chinese Academy of Forestry, Beijing, 100091 China; 2 Co-Innovation Center for Sustainable Forestry in Southern China, Nanjing Forestry University, Nanjing, 210037 China; 3 Key Laboratory of Horticultural Plant Biology of Ministry of Education, College of Horticulture and Forestry Sciences, Huazhong Agricultural University, Wuhan, 430070 China

**Keywords:** Reference genes, MicroRNAs, mRNAs, qRT-PCR, Normalization, Development, Poplar

## Abstract

**Background:**

Quantitative reverse transcriptase polymerase chain reaction (qRT-PCR) is a rapid and sensitive approach to identify miRNA and protein-coding gene expression in plants. However, because of the specially designated reverse transcription and shorter PCR products, very few reference genes have been identified for the quantitative analysis of miRNA expression in plants, and different internal reference genes are needed to normalize the expression of miRNAs and mRNA genes respectively. Therefore, it is particularly important to select the suitable common reference genes for normalization of quantitative PCR of miRNA and mRNA.

**Results:**

In this study, a modified reverse transcription PCR protocol was adopted for selecting and validating universal internal reference genes of mRNAs and miRNAs. Eight commonly used reference genes, four stably expressed novel genes in *Populus tremula*, three small noncoding RNAs and three conserved miRNAs were selected as candidate genes, and the stability of their expression was examined across a set of 38 tissue samples from four developmental stages of poplar clone 84K (*Populus alba* × *Populus glandulosa*). The expression stability of these candidate genes was evaluated systematically by four algorithms: geNorm, NormFinder, Bestkeeper and DeltaCt. The results showed that *Eukaryotic initiation factor 4A* III (*EIF4A*) and *U6*-*2* were suitable for samples of the callus stage; *U6*-*1* and *U6*-*2* were best for the seedling stage; *Protein phosphatase 2A*-*2* (*PP2A*-*2*) and *U6*-*1* were best for the plant stage; and *Protein phosphatase 2A*-*2* (*PP2A*-*2*) and *Oligouridylate binding protein 1B* (*UBP*) were the best reference genes in the adventitious root (AR) regeneration stage.

**Conclusions:**

The purpose of this study was to identify the most appropriate reference genes for qRT-PCR of miRNAs and mRNAs in different tissues at several developmental stages in poplar. *U6*-*1*, *EIF4A* and *PP2A*-*2* were the three most appropriate reference genes for qRT-PCR normalization of miRNAs and mRNAs during the plant regeneration process, and *PP2A*-*2* and *UBP* represent the best reference genes in the AR regeneration stage of poplar. This work will benefit future studies of expression and function analysis of miRNAs and their target genes in poplar.

**Electronic supplementary material:**

The online version of this article (10.1186/s13007-019-0420-1) contains supplementary material, which is available to authorized users.

## Introduction

Given its high sensitivity, quantitative accuracy, low cost and specificity, quantitative reverse transcriptase polymerase chain reaction (qRT-PCR) has become the most common and widely used technique for quantifying miRNA expression and mRNA transcript levels among different tissues and experimental conditions in plants [1, 2]. However, the accuracy of qRT-PCR is easily affected by several factors, including the quality of RNA samples, reverse transcription efficiency, cDNA quality and amount, and differences in extraneous tissue and cell activities [2–4]. To avoid bias in qRT-PCR analysis, validation of suitable reference genes for data normalization is an elementary prerequisite for each experimental condition in different tissues or species [5]. However, no single reference gene can be universal under all experimental situations, even including the most stable reference gene(s) reported [6, 7]. Therefore, optimal reference genes should be validated for different species, tissues or specific treatments.

MicroRNAs (miRNAs) are endogenous ~ 22 nt small noncoding RNAs that guide the cleavage or repress the translation of their target mRNAs by approximate base-pairing rules [8, 9] or mediate mRNA decay by directing rapid deadenylation of mRNAs [10]. In plants, miRNAs are master regulators in controlling developmental processes and in response to biotic and abiotic stress responses [11–15]. Due to its short sequence (only ~ 22 nt in length), the quantification of miRNAs by qRT-PCR requires extending the length of mature miRNAs using stem-loop primers [7, 16] or adding poly(A)-tails [17–20]. This extension requirement causes different internal reference genes to be commonly used for normalization in qRT-PCR of miRNAs and mRNAs. Some house-keeping genes, such as *actin 7* (*ACT 7*), eukaryotic initiation factor 4A III (*EIF4A*), *polyubiquitin* (*UBQ*), *glyceraldehyde*-*3*-*phosphate dehydrogenase* (*GAPDH*) and *protein phosphatase 2A*-*2* (*PP2A*-*2*), were widely adopted for gene expression analysis in diverse plants as reference genes [21–23]. Several noncoding RNA and small RNA, such as 5.8S ribosomal RNA (5.8S rRNA) and U6 small nuclear RNA (U6 snRNA), are commonly used as reference genes for miRNA quantity [24, 25]. Hurteau developed a modified universal reverse transcription PCR protocol, in which mature miRNAs could be polyadenylated by poly (A) polymerase and reverse transcribed into cDNA using oligo-dT primers [17]. Then, mRNAs and miRNAs could be specifically amplified and quantified at same transcriptional level, and the relative quantification of a miRNA and its predicted mRNA target can be both assessed precisely [17]. In this case, it is particularly important to select a suitable reference gene for normalization in quantitative PCR of miRNA and mRNA.

As a typical model woody plant, *Populus* has many advantages in basic research, such as rapid and perennial growth, moderate genome size, biomass-related traits and facile transformation [26]. Completion of the genomic sequence for *Populus trichocarpa* (black cottonwood) [27] has led to the development of genomic and molecular resources, and the ideal genetic transformation system provides a powerful genetic analysis tool for dissecting adaptive traits in poplar [28, 29]. Poplar clone 84K (*Populus alba* × *Populus glandulosa*) is now commonly used for gene functional studies because it is easier to obtain transgenic plants through *Agrobacterium tumefaciens*-mediated leaf discs [30–32]. The regeneration of transgenic plants involves callus induction, shoot differentiation, seedling culture and plant growth. This process is time consuming, requiring 2–3 months for tissue culture seedling and approximately 3–4 months for plant growth in soil. Therefore, we perform transgenic identification and gene expression analysis of early regenerated shoots and/or roots even small seedlings to reduce the time for identifying gene function in transgenic plants. In addition, the expression levels of miRNAs or target genes in different tissues are also required for analysis between transgenic and normal plants. However, most miRNA and mRNA expression levels vary greatly in different developmental stages and tissues, so it is necessary to identify a more stably expressed miRNA or gene as the internal reference to normalize expression using qRT-PCR.

In this study, we have tested 18 genes and noncoding RNAs for candidate reference genes. The expression stability of these genes was validated across a set of 38 tissue samples from four developmental biological processes of 84K poplar using a modified universal reverse transcription PCR protocol [17]. The cycle threshold (Ct) values of candidates were used to evaluate the expression stability using four algorithms: geNorm, NormFinder, Bestkeeper and DeltaCt. *U6*-*1*, *EIF4A* and *PP2A*-*2* were the top three most appropriate reference genes for qRT-PCR of miRNAs and mRNAs (including miRNA target genes) during the plant regeneration process, and *PP2A*-*2* and *UBP* were the best combination as reference genes in the AR regeneration stage of poplar.

## Results

### Verification of amplification and efficiency of the primers

A total of 12 protein-coding genes and 6 small noncoding RNAs were used as candidate reference genes for quantitative detection of miRNAs and mRNAs. The qRT-PCR primer sequences and amplicon characteristics of these candidate genes in 84K poplar are presented in Table 1. The PCR amplification specificities were confirmed by melting curves (Additional file 1: Fig. S1), agarose gel electrophoresis (Additional file 1: Fig. S2) and sequencing (Additional file 2: Fig. S3), which demonstrated the specific product of expected size and sequence. The qRT-PCR products ranged from 49 to 147 bp, and the sequence similarity between 84K poplar and *Populus trichocarpa* ranged from 97 to 100% despite belonging to different species. Therefore, the primers of these reference genes could also be used in other poplar species. To evaluate the amplification efficiency of pair-primers, the standard curves were obtained using a set of 10-fold diluted cDNA templates. The amplification efficiency (E) of the 18 candidate reference genes ranged from 96.16 to 116.69% and the regression coefficient (R^2^) varied between 0.941 and 1.000 (Table 1). These results suggest that the primers of all the candidate reference genes exhibit high amplification efficiency and specificity in the qRT-PCR system.

**Table 1:** The description of candidate reference genes and primers used in this study

Gene symbol	Gene name	Gene ID	Arabidopsis homolog	Forward primer sequence (5′–3′)	Reverse primer sequence (5′–3′)	Size (bp)	E (%)	R^2^
*ACT7*	Actin 7	Potri.001G309500	AT5G09810	GCATCCACGAGACTACATACAACTCA	GTGATCTCCTTGCTCATTCGGTCA	136	100.02	0.998
*EIF4A*	Eukaryotic initiation factor 4A	Potri.005G093900	AT3G19760	TACATTCATCGAATTGGTCGTTCTGGT	TTCATAGGCATTTCGTCAATCTGGG	137	101.50	0.995
*GAPDH*	Glyceraldehyde-3-phosphate dehydrogenase	Potri.012G094100	AT1G13440	AACCGACTTCATTGGTGACAACCG	CCACTCATTGTCATACCACGCAAC	106	100.72	0.997
*Histone*	Histone superfamily protein	Potri.005G072300	AT4G40030	ACTGTTGCTCTTCGTGAAATCCGTA	CTTAAAATCCTGGGCAATTTCACGAAC	105	96.62	0.998
*PP2A*-*2*	Protein phosphatase 2A-2	Potri.015G068300	AT1G10430	ACAGTTCAACCACACTAATGGGCTC	TTTGGCGCACTGAACACTGTAACCAC	114	103.41	0.988
*PP2A*-*A2*	Protein phosphatase 2A subunit A2	Potri.010G127500	AT3G25800	ATGAATTTCCTGATGTGCGACT	CAATGCCTATCCTCTGCAAGCTC	127	99.23	0.998
*RPS18*	Ribosomal protein S18	Potri.006G170500	AT1G07210	AGGCTCATCATCTTATCAAATCCCT	TCAATGCCACCAAATATTCGTTGCT	127	103.69	0.990
*UBQ10*	Polyubiquitin 10	Potri.001G418500	AT4G05320	GTTGATTTTTGCTGGGAAGC	GATCTTGGCCTTCACGTTGT	192	98.18	1.000
*ATPase*	ATP synthase subunit B	Potri.004G177500	AT4G38510	ACTCATCCCACCCCTGATCTTACGG	ACCAATGGCACTCTTCATGAGACGA	138	101.65	0.995
*UBP*	Oligouridylate binding protein 1B	Potri.006G279600	AT1G17370	GGCTTTGTTTCATTCCGTAATCAGCA	AACACCTTTAGTTGCCCAATTGCAT	111	100.92	0.978
*bHLH*	bHLH transcription factor	Potri.011G132400	AT5G54680	ATCTGAATCGTGTAGTGCGTCTAGCTC	GCATCAACCAAAATAGCAGCCTTGTCC	147	101.61	0.985
*DNAJ*	DNAJ homologue 2	Potri.010G243100	AT5G22060	AGGCAATTAATGACAAGGACCGTT	AGCCTCTCCAGGGAAAGTAATCCTC	131	101.98	0.996
*U6*-*1*	Small nuclear ribonucleoprotein family protein	Potri.001G166600	AT3G14080	GTGACCTTTATTGCGACATCCACT	CTTCTGAAACACGAGTCATATGTGGT	123	96.16	0.995
*U6*-*2*	Small nuclear ribonucleoprotein family protein	Potri.008G078400	AT2G43810	GCCTGTTGTGGTTAAGCTCAATTCTG	TTCCTCGTATGAAAGCATCACCAT	149	99.12	0.999
*5.8s*	5.8S ribosomal RNA gene	AJ006440		ACGTCTGCCTGGGTGTCAC	TCAACCACCGCTCGTCGTG	145	108.37	0.993
miR171	MIMAT0001985	ptc-miR171c		AGATTGAGCCGCGCCAATATC	AACGAGACGACGACAGACTTT	49	107.44	0.967
miR403	MIMAT0002056	ptc-miR403a		CGCGTTAGATTCACGCACAAACTC	AACGAGACGACGACAGACTTT	57	115.04	0.982
miR482	MIMAT0002103	ptc-miR482a.1		CCTACTCCTCCCATTCCAAAA	AACGAGACGACGACAGACTTT	50	116.69	0.941

### Expression profile of candidate reference genes during plant regeneration

**Fig. 1:**
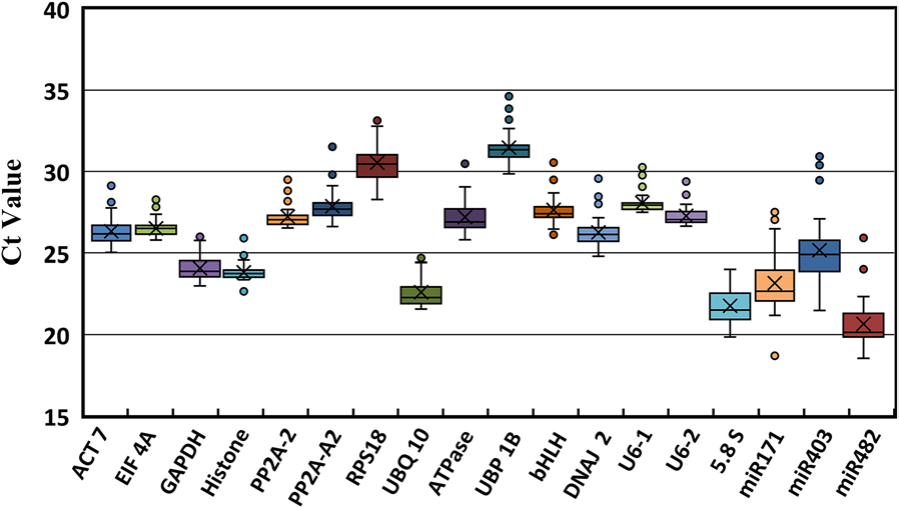
Box plots of the Ct values of 18 candidate reference genes during plant regeneration. The box indicates the 25th and 75th percentiles, and the whisker caps represent the maximum and minimum values. The line across the box indicates the median, and the cross depicts the mean

To evaluate the stability of the reference genes across all experimental samples, the transcript abundance of the 18 candidate reference genes was assessed based on mean Ct values. The average Ct values for the 18 candidate reference genes ranged from 21 to 33, with most values between 26 and 27 across all samples. miR482 had the highest expression level with a Ct value of 20.65 cycle, whereas *UBP* was the lowest abundantly expressed gene with Ct values up to 31.47 (Fig. 1). The Ct values of *EIF4A* (26.50 ± 0.55) and *U6*-*2* (27.27 ± 0.59) with minimum SD data indicate that these genes are the most stable genes in all the samples. The next most stable genes include *Histone* (23.83 ± 0.62) and *U6*-*1* (28.07 ± 0.62). Genes with more variable expression levels include miR403 (25.16 ± 2.29) and miR171 (23.16 ± 2.00) (Additional file 3: Table S1).

### Expression stability of candidate reference genes during plant regeneration

To detect the expression stability of 18 candidate reference genes more accurately, four software programs, including geNorm, NormFinder, BestKeeper and Delta-Ct method, were used for statistical analysis. These candidate reference genes were evaluated by each program and ranked from the most to the least stably expressed genes. Then, the geometric mean of each gene was calculated and reordered using RankAggreg software. Data of each reference gene from samples of different developmental stages were analyzed separately and then integrated together.

**Fig. 2:**
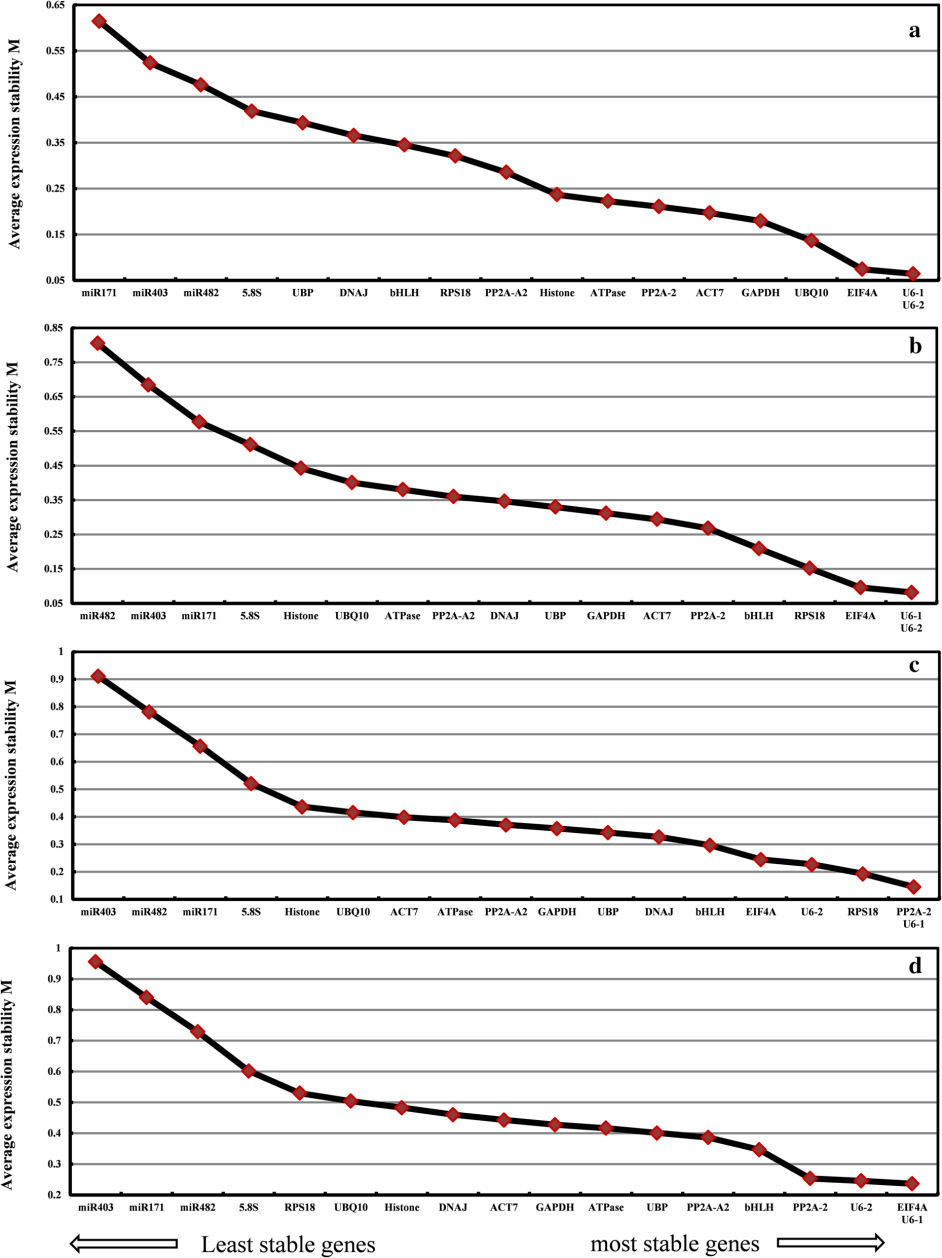
GeNorm analysis of average expression stability values (M) and ranking of the 18 candidate reference genes during plant regeneration. **a** Callus stage, **b** seedling stage, **c** plant stage, **d** ALL samples. A lower value of average expression stability indicates more stable expression

**Fig. 3:**
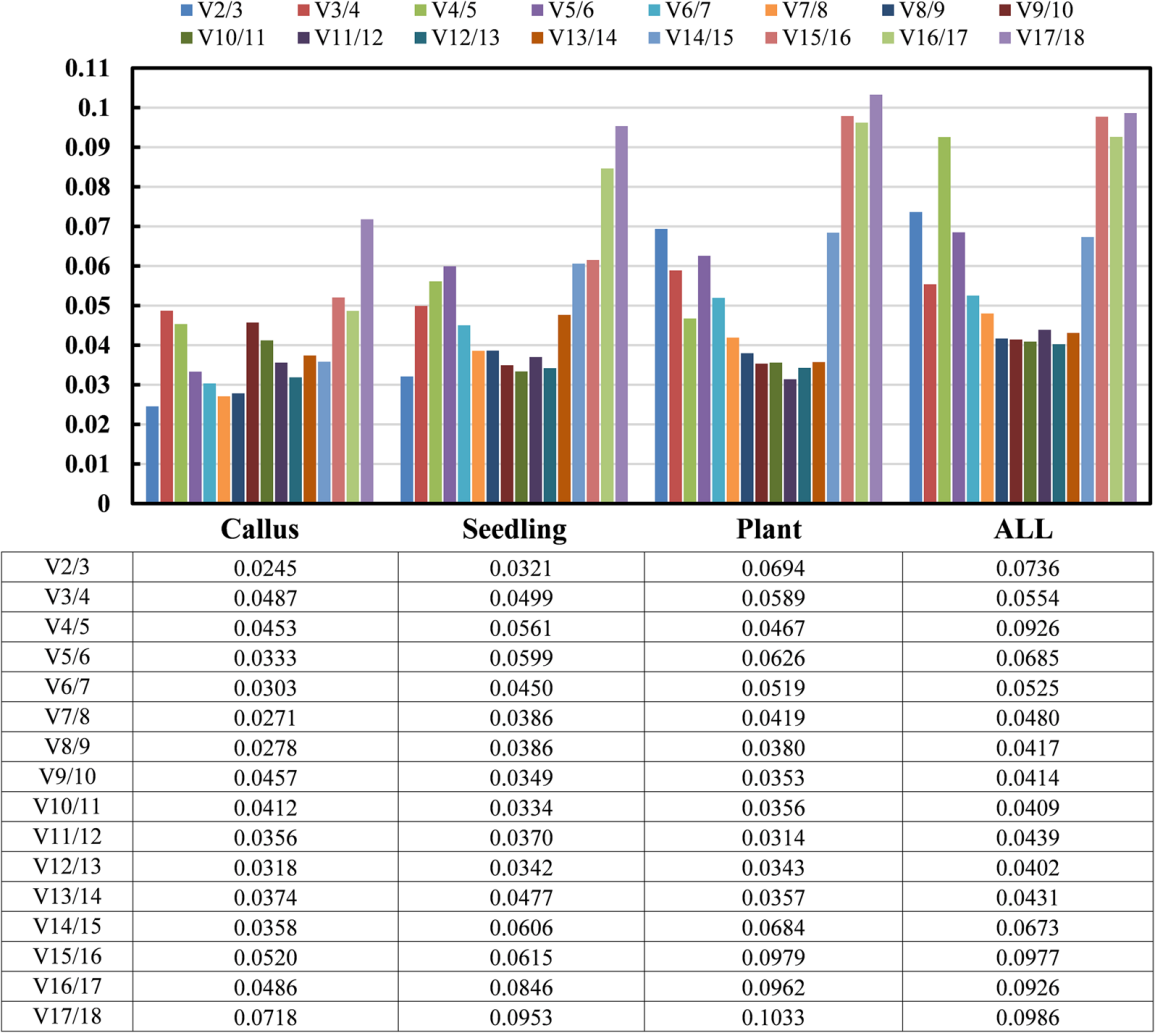
The optimal number of reference genes for accurate normalization calculated by geNorm during plant regeneration. Pairwise variation (V_n_/V_n+1_) analysis of 18 candidate reference genes analyzed in four sample subsets. callus, callus and shoots induced by 84K leaves; seedling, various tissues from 84K seedling stage; plant, various tissues from 84K plant stage; ALL, all samples from callus, seedling and plant developmental stages

The M-values of 18 candidate reference genes calculated by geNorm were all less than 1.5 in different tissues at plants developmental stages. *U6*-*1* and *U6*-*2* exhibited the highest stable expression with least M-values of 0.064 (Fig. 2a) and 0.082 (Fig. 2b) at the callus and seedling stage, respectively. *PP2A*-*2* and *U6*-*1* both exhibited the highest stable expression with the lowest M-value of 0.1456 at the plant stage (Fig. 2c), and *EIF4A* and *U6*-*1* exhibited the lowest M-value at 0.236 in all samples of these three stages (Fig. 2d). Overall, *U6*-*1* and *EIF4A* could be chosen as the most sable reference genes because of their lower M-value in various tissues at different developmental stages, whereas miR403 and miR171 were the least stable genes with increased M-values (from 0.577 to 0.955) in all samples. In the subsets of different developmental stages, all the pairwise variation values of V2/3 were less than 0.15, which suggests that the combined use of the two most stable reference genes would be most effective for normalizing gene expression analysis (Fig. 3).

The stability values of the candidate reference genes constructed by NormFinder are presented in Table 2. At the callus stage, *Histone* was the most stable gene with the lowest stability value followed by *EIF4A* and *U6*-*2*. *U6*-*1*, *U6*-*2* and *EIF4A* exhibited more stable expression at the seedling stage, whereas *UBP*, *bHLH* and *DNAJ* exhibited increased stability in various tissues at the plant stage. In all samples, *bHLH*, *PP2A*-*2* and *U6*-*1* were the top three stable genes. miR403, miRN482 and miR171 were the least stable genes during the plant regeneration process.

**Table 2:** Ranking of the expression stability of candidate reference genes calculated using NormFinder during plant regeneration

Rank	Callus		Seedlings		Plants		Total	
	Gene	Stability	Gene	Stability	Gene	Stability	Gene	Stability
1	*Histone*	0.041	*U6*-*1*	0.041	*UBP*	0.210	*bHLH*	0.275
2	*EIF4A*	0.168	*U6*-*2*	0.041	*bHLH*	0.228	*PP2A*-*2*	0.302
3	*U6*-*2*	0.213	*EIF4A*	0.043	*DNAJ*	0.247	*U6*-*1*	0.303
4	*U6*-*1*	0.221	*RPS18*	0.082	*U6*-*1*	0.288	*UBP*	0.308
5	*PP2A*-*2*	0.222	*bHLH*	0.151	*PP2A*-*2*	0.296	*U6*-*2*	0.376
6	*ACT7*	0.277	*GAPDH*	0.267	*RPS18*	0.380	*EIF4A*	0.402
7	*ATPase*	0.284	*ACT7*	0.282	*PP2A*-*A2*	0.418	*GAPDH*	0.419
8	*UBQ10*	0.310	*PP2A*-*2*	0.288	*UBQ10*	0.424	*PP2A*-*A2*	0.427
9	*GAPDH*	0.335	*DNAJ*	0.374	*EIF4A*	0.427	*DNAJ*	0.461
10	*RPS18*	0.417	*UBP*	0.392	*Histone*	0.440	*Histone*	0.485
11	*5.8S*	0.424	*PP2A*-*A2*	0.472	*ACT7*	0.468	*ATPase*	0.499
12	*bHLH*	0.428	*Histone*	0.508	*U6*-*2*	0.471	*ACT7*	0.521
13	*PP2A*-*A2*	0.441	*UBQ10*	0.558	*GAPDH*	0.471	*RPS18*	0.561
14	*UBP*	0.526	*ATPase*	0.575	*ATPase*	0.544	*UBQ10*	0.582
15	*DNAJ*	0.533	*5.8S*	0.855	*5.8S*	0.921	*5.8S*	0.967
16	*miR482*	0.712	*miR171*	0.901	*miR171*	1.405	*miR171*	1.473
17	*miR403*	0.773	*miR403*	1.498	*miR482*	1.618	*miR482*	1.506
18	*miR171*	1.288	*miR482*	1.710	*miR403*	1.852	*miR403*	1.767

The most stable genes based on BestKeeper analysis exhibiting the lowest CV ± SD were *EIF4A* (0.28 ± 0.07), *Histone* (1.14 ± 0.27) and *EIF4A* (1.63 ± 0.43) in various tissues at the callus, seedling and plant stages, respectively (Table 3). *U6*-*1* and *U6*-*2* exhibited stable expression during all these developmental stages. In all samples, *EIF4A* (1.39 ± 0.37) was the most stable gene followed by *U6*-*1* (1.38 ± 0.39) and *Histone* (1.65 ± 0.39), whereas miR403, miR171 and miR482 were the most unstable genes with highest SV ± SD and a SD value greater than 1.

**Table 3:** Ranking of the expression stability of candidate reference genes calculated by BestKeeper during plant regeneration

Rank	Callus			Seedlings			Plants			Total		
	Gene	SD	CV	Gene	SD	CV	Gene	SD	CV	Gene	SD	CV
1	*EIF4A*	0.07	0.28	*Histone*	0.27	1.14	*EIF4A*	0.43	1.63	*EIF4A*	0.37	1.39
2	*U6*-*2*	0.08	0.31	*U6*-*1*	0.28	1	*U6*-*2*	0.49	1.77	*U6*-*1*	0.39	1.38
3	*U6*-*1*	0.12	0.41	*EIF4A*	0.28	1.09	*U6*-*1*	0.52	1.83	*Histone*	0.39	1.65
4	*UBQ10*	0.15	0.65	*U6*-*2*	0.29	1.09	*Histone*	0.53	2.2	*U6*-*2*	0.44	1.6
5	*Histone*	0.16	0.65	*RPS18*	0.37	1.22	*PP2A*-*2*	0.54	1.99	*PP2A*-*2*	0.49	1.79
6	*ACT7*	0.17	0.65	*bHLH*	0.4	1.48	*GAPDH*	0.58	2.4	*bHLH*	0.61	2.21
7	*ATPase*	0.17	0.63	*miR171*	0.44	1.91	*RPS18*	0.6	1.94	*GAPDH*	0.61	2.54
8	*GAPDH*	0.19	0.79	*GAPDH*	0.45	1.89	*bHLH*	0.69	2.48	*UBP*	0.66	2.1
9	*PP2A*-*2*	0.19	0.69	*PP2A*-*2*	0.49	1.82	*PP2A*-*A2*	0.71	2.53	*PP2A*-*A2*	0.66	2.38
10	*PP2A*-*A2*	0.32	1.15	*DNAJ*	0.49	1.96	*DNAJ*	0.71	2.65	*ACT7*	0.7	2.64
11	*bHLH*	0.33	1.22	*ACT7*	0.56	2.19	*ATPase*	0.75	2.74	*UBQ10*	0.7	3.1
12	*5.8S*	0.33	1.5	*UBQ10*	0.57	2.55	*UBP*	0.76	2.39	*DNAJ*	0.71	2.69
13	*RPS18*	0.34	1.15	*UBP*	0.6	1.94	*ACT7*	0.81	3.08	*ATPase*	0.74	2.7
14	*DNAJ*	0.42	1.62	*PP2A*-*A2*	0.6	2.21	*UBQ10*	0.84	3.74	*RPS18*	0.76	2.48
15	*UBP*	0.44	1.4	*ATPase*	0.63	2.36	*5.8S*	1.09	5.03	*5.8S*	0.93	4.25
16	*miR482*	0.48	2.39	*5.8S*	0.79	3.62	*miR482*	1.27	6.11	*miR482*	1.11	5.39
17	*miR403*	0.64	2.75	*miR403*	0.83	3.36	*miR171*	1.82	7.68	*miR171*	1.48	6.39
18	*miR171*	0.88	4.14	*miR482*	1.22	5.8	*miR403*	1.97	7.62	*miR403*	1.63	6.47

The rankings using the Delta Ct method are presented in Table 4. *EIF4A*, *U6*-*2* and *U6*-*1* were the top three stable genes at the callus and seedling stages. *PP2A*-*2* was the most stable gene at the plant stage and in all samples during these three developmental stages, followed by *U6*-*1*. This finding indicates that *EIF4A*, *PP2A*-*2* and *U6*-*1* might be the most stably expressed genes as determined using the Delta Ct method.

**Table 4:** Expression stability ranking of the 18 candidate reference genes during plant regeneration

Method	1	2	3	4	5	6	7	8	9
(A) Ranking order across tissues at callus stage (better–good–average)									
Genorm	*U6*-*1|U6*-*2*		*EIF4A*	*UBQ10*	*GAPDH*	*ACT7*	*PP2A*-*2*	*ATPase*	*Histone*
Normfinder	*Histone*	*EIF4A*	*U6*-*2*	*U6*-*1*	*PP2A*-*2*	*ACT7*	*ATPase*	*UBQ10*	*GAPDH*
BestKeeper	*EIF4A*	*U6*-*2*	*U6*-*1*	*UBQ10*	*Histone*	*ACT7*	*ATPase*	*GAPDH*	*PP2A*-*2*
Delta CT	*EIF4A*	*U6*-*2*	*U6*-*1*	*Histone*	*PP2A*-*2*	*ATPase*	*ACT7*	*UBQ10*	*GAPDH*
RankAggreg	*EIF4A*	*U6*-*2*	*U6*-*1*	*Histone*	*UBQ10*	*ACT7*	*PP2A*-*2*	*ATPase*	*GAPDH*
(B) Ranking order across tissues at seedlings stage (better–good–average)									
Genorm	*U6*-*1|U6*-*2*		*EIF4A*	*RPS18*	*bHLH*	*PP2A*-*2*	*ACT7*	*GAPDH*	*UBP*
Normfinder	*U6*-*1*	*U6*-*2*	*EIF4A*	*RPS18*	*bHLH*	*GAPDH*	*ACT7*	*PP2A*-*2*	*DNAJ*
BestKeeper	*Histone*	*U6*-*1*	*EIF4A*	*U6*-*2*	*RPS18*	*bHLH*	miR171	*GAPDH*	*PP2A*-*2*
Delta CT	*U6*-*2*	*U6*-*1*	*EIF4A*	*PP2A*-*2*	*RPS18*	*bHLH*	*ACT7*	*GAPDH*	*UBP*
RankAggreg	*U6*-*1*	*U6*-*2*	*EIF4A*	*RPS18*	*bHLH*	*PP2A*-*2*	*ACT7*	*GAPDH*	*DNAJ*
(C) Ranking order across tissues at plants stage (better–good–average)									
Genorm	*PP2A*-*2|U6*-*1*		*RPS18*	*U6*-*2*	*EIF4A*	*bHLH*	*DNAJ*	*UBP*	*GAPDH*
Normfinder	*UBP*	*bHLH*	*DNAJ*	*U6*-*1*	*PP2A*-*2*	*RPS18*	*PP2A*-*A2*	*UBQ10*	*EIF4A*
BestKeeper	*EIF4A*	*U6*-*2*	*U6*-*1*	*Histone*	*PP2A*-*2*	*GAPDH*	*RPS18*	*bHLH*	*PP2A*-*A2*
Delta CT	*PP2A*-*2*	*U6*-*1*	*bHLH*	*UBP*	*DNAJ*	*RPS18*	*PP2A*-*A2*	*EIF4A*	*GAPDH*
RankAggreg	*U6*-*1*	*PP2A*-*2*	*bHLH*	*RPS18*	*EIF4A*	*U6*-*2*	*UBP*	*DNAJ*	*PP2A*-*A2*
(D) Ranking order under all samples (better–good–average)									
Genorm	*EIF4A|U6*-*1*		*U6*-*2*	*PP2A*-*2*	*bHLH*	*PP2A*-*A2*	*UBP*	*ATPase*	*GAPDH*
Normfinder	*bHLH*	*PP2A*-*2*	*UBP*	*U6*-*1*	*U6*-*2*	*EIF4A*	*GAPDH*	*PP2A*-*A2*	*DNAJ*
BestKeeper	*EIF4A*	*U6*-*1*	*Histone*	*U6*-*2*	*PP2A*-*2*	*bHLH*	*GAPDH*	*UBP*	*PP2A*-*A2*
Delta CT	*PP2A*-*2*	*U6*-*1*	*bHLH*	*U6*-*2*	*UBP*	*EIF4A*	*PP2A*-*A2*	*GAPDH*	*ATPase*
RankAggreg	*U6*-*1*	*EIF4A*	*PP2A*-*2*	*U6*-*2*	*UBP*	*PP2A*-*A2*	*bHLH*	*GAPDH*	*Histone*

Method	10	11	12	13	14	15	16	17	18
(A) Ranking order across tissues at callus stage (better–good–average)									
Genorm	*PP2A*-*A2*	*RPS18*	*bHLH*	*DNAJ*	*UBP*	*5.8S*	miR482	miR403	miR171
Normfinder	*RPS18*	*5.8S*	*bHLH*	*PP2A*-*A2*	*UBP*	*DNAJ*	miR482	miR403	miR171
BestKeeper	*PP2A*-*A2*	*bHLH*	*5.8S*	*RPS18*	*DNAJ*	*UBP*	miR482	miR403	miR171
Delta CT	*PP2A*-*A2*	*RPS18*	*bHLH*	*5.8S*	*DNAJ*	*UBP*	miR482	miR403	miR171
RankAggreg	*RPS18*	*PP2A*-*A2*	*bHLH*	*5.8S*	*DNAJ*	*UBP*	miR482	miR403	miR171
(B) Ranking order across tissues at seedlings stage (better–good–average)									
Genorm	*DNAJ*	*PP2A*-*A2*	*ATPase*	*UBQ10*	*Histone*	*5.8S*	miR171	miR403	miR482
Normfinder	*UBP*	*PP2A*-*A2*	*Histone*	*UBQ10*	*ATPase*	*5.8S*	miR171	miR403	miR482
BestKeeper	*DNAJ*	*ACT7*	*UBQ10*	*UBP*	*PP2A*-*A2*	*ATPase*	*5.8S*	miR403	miR482
Delta CT	*DNAJ*	*PP2A*-*A2*	*ATPase*	*UBQ10*	*Histone*	*5.8S*	miR171	miR403	miR482
RankAggreg	*UBP*	*PP2A*-*A2*	*Histone*	*UBQ10*	*ATPase*	*5.8S*	miR171	miR403	miR482
(C) Ranking order across tissues at plants stage (better–good–average)									
Genorm	*PP2A*-*A2*	*ATPase*	*ACT7*	*UBQ10*	*Histone*	*5.8S*	miR171	miR482	miR403
Normfinder	*Histone*	*ACT7*	*U6*-*2*	*GAPDH*	*ATPase*	*5.8S*	miR171	miR482	miR403
BestKeeper	*DNAJ*	*ATPase*	*UBP*	*ACT7*	*UBQ10*	*5.8S*	miR482	miR171	miR403
Delta CT	*U6*-*2*	*ACT7*	*UBQ10*	*ATPase*	*Histone*	*5.8S*	miR171	miR482	miR403
RankAggreg	*GAPDH*	*Histone*	*ACT7*	*ATPase*	*UBQ10*	*5.8S*	miR171	miR482	miR403
(D) Ranking order under all samples (better–good–average)									
Genorm	*ACT7*	*DNAJ*	*Histone*	*UBQ10*	*RPS18*	*5.8S*	miR482	miR171	miR403
Normfinder	*Histone*	*ATPase*	*ACT7*	*RPS18*	*UBQ10*	*5.8S*	miR171	miR482	miR403
BestKeeper	*ACT7*	*UBQ10*	*DNAJ*	*ATPase*	*RPS18*	*5.8S*	miR482	miR171	miR403
Delta CT	*ACT7*	*DNAJ*	*Histone*	*UBQ10*	*RPS18*	*5.8S*	miR171	miR482	miR403
RankAggreg	*ATPase*	*ACT7*	*DNAJ*	*UBQ10*	*RPS18*	*5.8S*	miR482	miR171	miR403

To obtain a consensus regarding the most stable reference genes as recommended by the four methods, the geometric mean of four algorithms corresponding rankings for each candidate gene were calculated using the RankAggreg software. *EIF4A*, *U6*-*2* and *U6*-*1* were ranked as the top three stable reference genes in samples from the callus developmental stage. *U6*-*1* was the most stable gene at the seedling and plant stages, and *U6*-*2* and *PP2A*-*2* ranked second at the seedling stage and plant stage respectively. The expression values of miR403, miR171 and miR482 were extremely variable in all tissues at different stages. Based on these results, *U6*-*1*, *EIF4A* and *PP2A*-*2* are the best combination of reference genes in all samples of different developmental stages. *EIF4A* and *U6*-*2* are the most stable reference genes for the samples from callus stage. *U6*-*1* and *U6*-*2* are the best reference genes for the seedling stage, and *U6*-*1* and *PP2A*-*2* suitable for the plant stage.

### Expression stability of the candidate reference genes in AR developmental stage

The expression profiles of the 18 candidate reference genes and the ranking of their expression stability in the AR developmental stage were different from those during the plant regeneration processes. With the exception of *5.8* *s* and miRNAs, the Ct values for the remaining 14 candidate reference genes in the AR developmental stage were relatively higher compared with the other stages having most values between 27 and 33. The Ct values of miR482 (24.62 ± 0.42) had a minimum SD value and *5.8s* (22.93 ± 4.70) had a maximum SD value (Additional file 3: Table S1). In geNorm analysis, *EIF4A* and *PP2A*-*2* exhibited the highest stability with the lowest M-value. The pairwise variation was 0.133 for V2/3 values. Thus, the combined use of the two most stable reference genes would be suitable for normalizing gene expression analysis. Using NormFinder software, *bHLH* was identified as the most stable gene with the lowest stability value followed by *UBP* and *PP2A*-*2*. *miR482* (1.34 ± 0.33), *PP2A*-*A2* (1.41 ± 0.45) and *UBP* (1.42 ± 0.47) exhibited more stability with lower CV ± SD calculated by BestKeeper. *PP2A*-*2*, *ACT7* and *UBP* were the top three stable genes calculated using the Delta Ct method. RankAggreg ranked *PP2A*-*2*, *UBP* and *bHLH* as the top three stable reference genes in the AR developmental stages. In contrast, *5.8s*, miR171, miR403 and *GAPDH* were unstable genes identified by all algorithms (Table 5).

**Table 5:** Ranking of candidate reference genes in order of their expression stability in the AR developmental stage

Ranking	Genorm		Normfinder		BestKeeper			Delta CT	RankAggreg
	Gene	Stability	Gene	Stability	Gene	SD	CV		
1	*EIF4A* *PP2A*-*2*	0.400	*bHLH*	0.422	miR482	0.33	1.34	*PP2A*-*2*	*PP2A*-*2*
2			*UBP*	0.424	*PP2A*-*A2*	0.45	1.41	*ACT7*	*UBP*
3	*RPS18*	0.432	*PP2A*-*2*	0.501	*UBP*	0.47	1.42	*UBP*	*bHLH*
4	*bHLH*	0.612	*U6*-*1*	0.517	*ATPase*	0.48	1.61	*bHLH*	*PP2A*-*A2*
5	*UBP*	0.669	*PP2A*-*A2*	0.576	*ACT7*	0.56	1.85	*U6*-*1*	*ACT7*
6	*U6*-*1*	0.704	*U6*-*2*	0.671	*bHLH*	0.61	2.03	*PP2A*-*A2*	miR482
7	*ATPase*	0.749	*miR482*	0.792	*U6*-*1*	0.67	2.08	miR482	*U6*-*1*
8	*PP2A*-*A2*	0.781	*ACT7*	0.830	*PP2A*-*2*	0.69	2.13	*ATPase*	*EIF4A*
9	*ACT7*	0.804	*EIF4A*	0.931	*RPS18*	0.81	2.51	*EIF4A*	*ATPase*
10	miR482	0.843	*ATPase*	0.934	*Histone*	0.83	2.73	*U6*-*2*	*RPS18*
11	*Histone*	0.877	*Histone*	0.951	*U6*-*2*	0.88	2.77	*Histone*	*U6*-*2*
12	*U6*-*2*	0.904	*RPS18*	1.054	*UBQ10*	1.00	3.55	*DNAJ*	*Histone*
13	*DNAJ*	0.941	*DNAJ*	1.078	*DNAJ*	1.03	3.23	*UBQ10*	*DNAJ*
14	*GAPDH*	0.963	*UBQ10*	1.160	*EIF4A*	1.04	3.32	*RPS18*	*UBQ10*
15	*UBQ10*	1.008	*GAPDH*	1.338	*GAPDH*	1.15	3.85	*GAPDH*	*GAPDH*
16	miR171	1.062	miR403	1.508	miR403	1.22	4.54	miR403	miR403
17	miR403	1.136	miR171	1.979	miR171	1.34	6.68	miR171	miR171
18	*5.8S*	1.589	*5.8S*	4.693	*5.8S*	3.69	16.07	*5.8S*	*5.8S*

### Reference genes validation

**Fig. 4:**
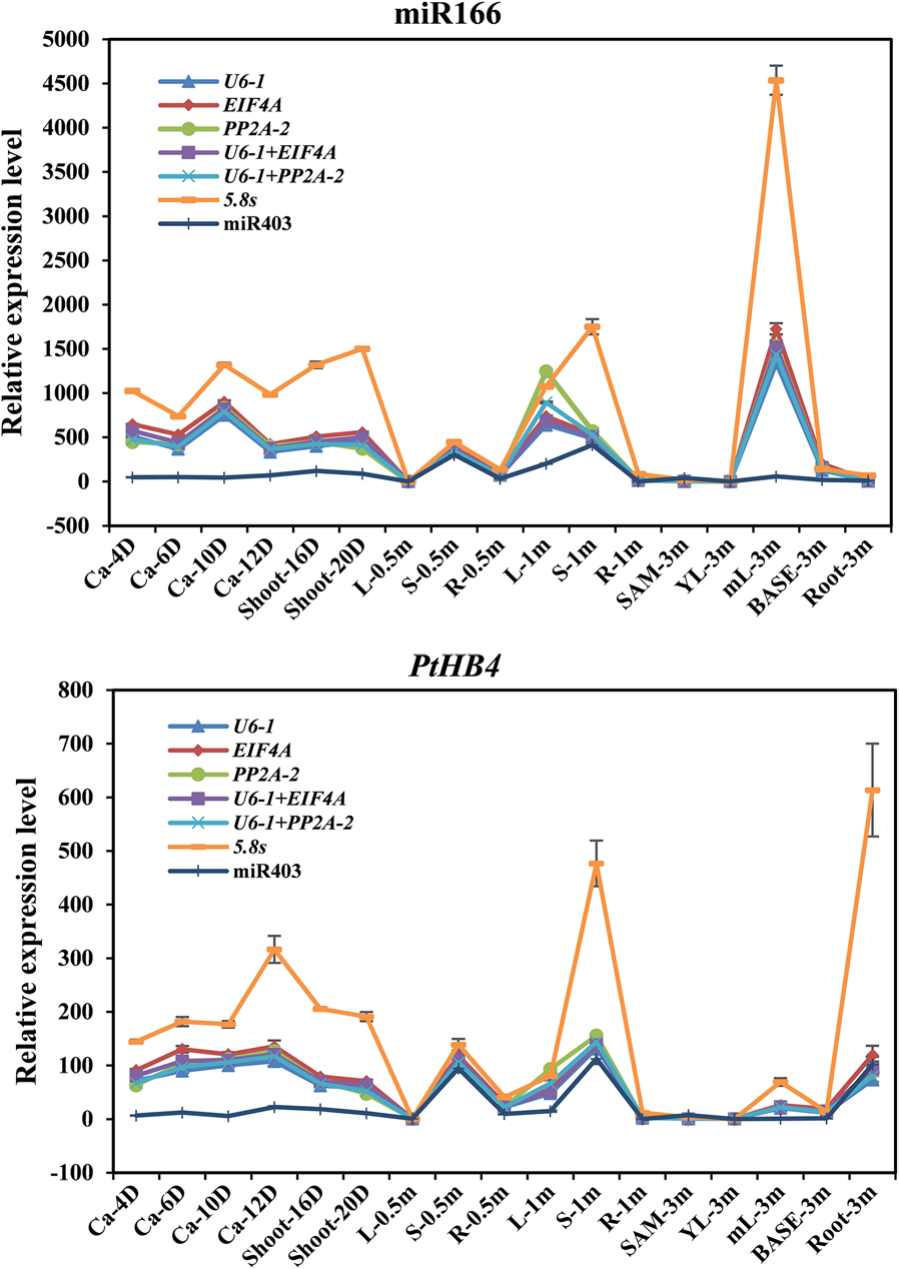
Relative expression of miR166 and *PtHB4* using validated reference genes for normalization under plant regeneration process. The validated reference gene(s) used as normalization factors were one (*U6*-*1*, *EIF4A*, *PP2A*-*2* alone) or two (*U6*-*1* + *EIF4A* and *U6*-*1* + *PP2A*-*2*) of three best stable reference genes and two most unstable reference genes (*5.8s* and miR403)

To validate the stability of the above reference genes, the expression profiles of miR166a, which is highly expressed in leaves and xylem [33], and *PtHB4*, which expressed specifically in shoot apex, cambium and xylem [34, 35], were measured and normalized with the most stable and the least stable reference genes. The three top ranking reference genes (*U6*-*1*, *EIF4A*, *PP2A*-*2*) either alone or combination and two unstable reference genes (5.8 s and miR403), were used as reference genes for RT-qPCR analysis at callus, seedling and plant stages. As shown in Fig. 4, the relative expression profiles of miR166a and *PtHB4* normalized with *U6*-*1*, *EIF4A*, *PP2A*-*2*, *U6*-*1* + *EIF4A* and *U6*-*1* + *PP2A*-*2* exhibited perfect consistency in seventeen tissues. Compared with tender leaves from 0.5-month-old seedlings, miR166a was highly expressed in stems, mature leaves and all samples at the callus stage (Fig. 4), whereas *PtHB4* was specifically expressed in stems and samples at the callus stage (Fig. 4). However, the expression values of miR166a and *PtHB4* normalized with 5.8s were increased, and the values normalized to miR403 were reduced compared with than other reference genes (Fig. 4). For example, the relative expression value of miR166 in mature leaves at 3 months was approximately 1500 when normalized with *U6*-*1*. However, the expression value was 4500 when normalized with *5.8* *s* and 18 when normalized with miR403.

**Fig. 5:**
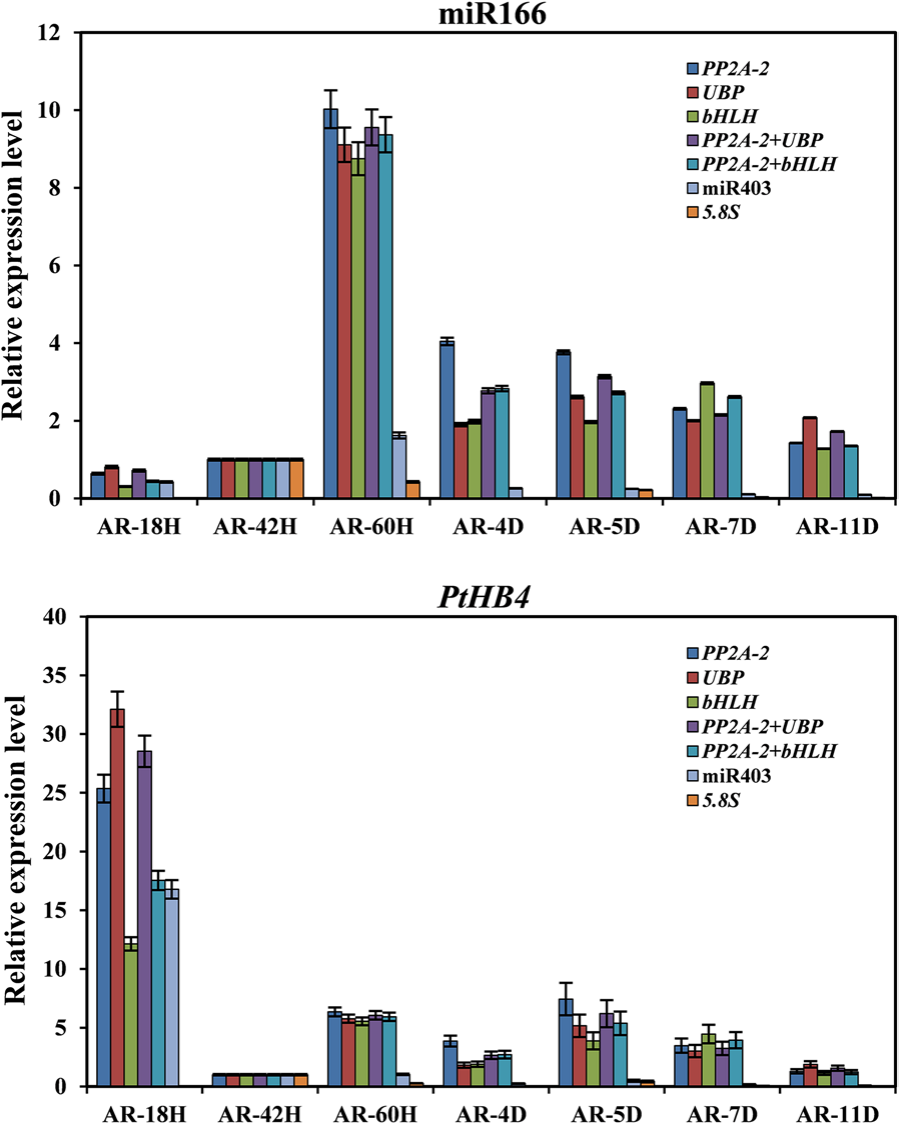
Relative expression of miR166 and *PtHB4* using validated reference genes for normalization under AR regeneration. *PP2A*-*2*, *UBP*, *bHLH*, *PP2A*-*2* + *UBP* and *PP2A*-*2* + *bHLH* were selected for the stable reference genes, and 5.8 s and miR403 were the two least stable genes for normalization RT-qPCR analysis

Because the combined use of two most stable reference genes would be suitable for normalizing gene expression analysis at the AR developmental stage, *PP2A*-*2*, *UBP*, *bHLH*, *PP2A*-*2* + *UBP* and *PP2A*-*2* + *bHLH* were selected as stable reference genes, and 5.8s and miR403 were selected as the two least stable reference genes for RT-qPCR analysis of the AR developmental stage. As shown in Fig. 5, the relative expression values of miR166 and *PtHB4* normalized with *PP2A*-*2* were higher than values normalized with *bHLH*, but similar expression patterns were noted. Therefore, the combination of *PP2A*-*2* and *bHLH* could neutralize the expression values normalized with *PP2A*-*2* and *bHLH.* miR166 was highly expressed in AR-60H, in which the AR callus regenerated and expanded. *PtHB4* exhibited a higher expression value in AR-18H when AR induction had begun. The relative expression profiles of miR166 and *PtHB4* normalized with *5.8s* and miR403 were reduced compared with values normalized with other reference genes, and different expression patterns were noted (Fig. 5). Overall, the combination of, *PP2A*-*2* and *UBP* or *PP2A*-*2* and *bHLH* should be the best reference gene set for normalization of qRT-PCR at the AR developmental stage.

## Discussion

Numerous studies have performed reference gene validation experiments for mRNA and miRNA qRT-PCR [36, 37]. For example, some house-keeping genes, such as *ACT 7*, *UBQ*, *GAPDH* and *TUB*, were widely used for gene expression analysis of mRNAs in diverse plants, and several noncoding RNAs, such as *U6 snRNA* and *5.8S rRNA*, were typically chosen for normalizing miRNA quantification data. As miRNA research continues to expand, the potential use of miRNAs as reference genes has attracted increasing attention, and some small RNAs were obtained from plant species, such as grapevine [7], wheat [38], peach [39], soybean [40], and tea [41] demonstrating that miRNAs are more stable than the currently used reference genes under specific conditions. However, there are no reports on universal reference genes for the quantitative expression of both mRNA and miRNA, which may be necessary to calibrate both miRNA and its target gene(s) in a given sample. In general, the reverse transcription of miRNA is different from other types of RNA because of the shorter length of miRNA. Therefore, quantitative expression analysis of miRNAs and mRNAs requires different internal reference genes for normalization of their respective transcripts [15, 42]. If miRNA and mRNA expression data are normalized with universal internal reference genes, quantitative PCR must be performed at the same transcriptional level. Fortunately, Hurteau developed a modified universal reverse transcription PCR protocol that is designed to specifically amplify and quantify mRNAs and miRNAs from the same sample [17]. The modified technique involves the enzymatic addition of a poly A tail to non-poly(A) RNAs followed by reverse transcription using a universal RT-primer. Then, the transcript-specific forward primers can be used to amplify noncoding RNA (including miRNA) and mRNA from the same sample [17]. To obtain suitable universal reference genes for normalization of miRNA and mRNA expression, the expression stability of the selected candidate reference genes in this study was validated across 38 tissue samples from four developmental stages in 84K poplar using a modified universal reverse transcription PCR protocol.

Four algorithms (geNorm, NormFinder, DeltaCt, Bestkeeper) were used to minimize the bias for the evaluation of the 18 candidate reference genes. Discrepancy was observed in gene stability ranking and validation generated by the four different algorithms above. For example, at the plant stage, *PP2A*-*2* was ranked first by geNorm and DeltaCt, whereas it was ranked fifth by NormFinder and Bestkeeper. *Histone* was ranked among the top four stable genes by BestKeeper in all samples at the seedling and plant stages but was ranked in the middle or bottom position by geNorm, NormFinder and DeltaCt. This apparent divergence is probably due to the statistical algorithms used to calculate stability. Genorm and Normfinder software have similar algorithms, which calculate the ΔCt values and the stability of each internal reference gene according to the minimum Ct value in all the samples [43, 44]. Therefore, the alteration of the minimum Ct value will have a great influence on the stability of expression of candidate reference genes. Besides that, BestKeeper program can calculate the standard deviation (SD) and variation coefficient (CV) values between the Ct values of each internal reference gene and the average of all Ct values, so the stability of candidate reference gene depending on the dispersion degree of Ct values [45]. Thus, it has been recommended that more than two algorithms should be used for reference gene stability evaluation [43]. In this study, RankAggreg software was used to generate the final overall ranking of the tested reference genes based on the geometric mean of the weights of every gene calculated by each program. Through this comprehensive ranking analysis, *U6*-*1*, *EIF4A* and *PP2A*-*2* ranked as top three for all samples at the callus, seedling and plant stages. Thus, these genes would be the most suitable internal reference genes for the quantification of mRNAs and noncoding RNAs during the plant regeneration process of poplar.

However, the candidate reference genes selected for samples during the AR developmental stage were almost completely different. *UBP* ranked among the top three most stable genes in AR developmental stage but near the bottom at the callus stage using the four algorithms. In contrast, miR482 was the most stable gene in the AR developmental stage identified by Bestkeeper, but it was a highly unstable gene in other developmental stages. This is mainly because the development of adventitious roots is quite different from other biological processes, which include root primordium initiation, callus differentiation, adventitious root emergence and elongation process [46, 47]. During this process, tissue morphology and structure have undergone dramatic changes, and gene expression patterns varied tremendously [48–50]. Therefore, the genes expressed stably in other tissues or developmental stages may not have stable expression values during AR developmental stages. Comparatively, *PP2A*-*2*, *UBP* and *bHLH* exhibited more stable expression in the AR developmental stage; thus, these genes could be suitable for normalization. These results indicate that different sets of internal reference genes may be assigned for different tissues and developmental stages even in the same species. Furthermore, *5.8S*, which is a commonly used reference gene for miRNA, and commonly used miRNAs (miR171, miR482 and miR403) exhibited extremely unstable expression and were ranked among the four least stable genes during all developmental stages in poplar, indicating these miRNAs were more unstable than protein-coding genes and exhibited obvious tissue-specific expression.

Traditionally, reference genes are typically cellular maintenance genes that play housekeeping roles in basic cellular components and functions [23]. In this study, with the exception of two house-keeping genes (*EIF4A* and *PP2A*-*2*), two noncoding RNA (*U6*-*1* and *U6*-*2*) and a nontraditional reference gene (*UBP*) were also confirmed as the most suitable internal reference genes at the seedling and AR developmental stages. *EIF4A* and *PP2A* are typically used as reference genes for quantitative PCR and exhibit stable expression in different tissues, developmental stages or biotic and abiotic stress conditions in a number of species. For example, *PP2A* and *EIF4A* were reported as the best reference genes for all samples of various tissues and abiotic stress conditions in Sorghum [51], and *PP2A* was suitable for Switchgrass [52]. *EIF4α* was also ranked as a stably expressed gene under most of the experimental conditions tested in *Carica papaya* [53], different tissue/organs and fruit developmental stages in *Litsea cubeba* [54], and different tissues under abiotic stresses in *Pennisetum glaucum* [55]. *PP2A* housekeeping genes were superior references for normalization of gene expression data in different cotton plant organs [56], different color lines of cineraria during flower developmental stages [57] and diurnal and developmental time-course in lettuce [58]. In addition, *U6* is also one of the most commonly used reference genes in miRNA qRT-PCR and had the most stable expression in reference gene selection studies [7, 25, 41]. In our study, *U6*-*1* and *U6*-*2* exhibited the most stable expression values at the callus and seedling stages, and *U6*-*1* ranked among the top two at the plant stage. Therefore, as a noncoding RNA, *U6* can be used not only as a good reference gene for RT-qPCR of miRNA alone but also a universal internal reference gene for mRNA quantification. The *UBP* gene encodes a heterogeneous nuclear RNA binding protein (hnRNP) that is involved in the regulation of pre-mRNA maturation at different levels and pre-mRNA splicing [59]. *UBP* is not a traditional internal reference gene, but its expression value did not change in various tissues and developmental stages in poplar [60]. *UBP* was the most stably expressed gene in the AR regeneration stage, and the stability of *UBP* was ranked at the top and middle positions at the plant and seedling stages. This finding indicates that *UBP* might be a reliable reference gene used in the AR and plant developmental stages in poplar.

## Conclusion

The purpose of this study was to identify the most appropriate reference genes for qRT-PCR of miRNAs and mRNAs during poplar regeneration and development. The expression stability of 18 candidate genes was validated and evaluated across 38 tissue samples from four developmental stages of 84K poplar using four algorithms. The results demonstrated that *EIF4A* and *U6*-*2* were suitable for samples of callus stage, *U6*-*1* and *U6*-*2* were best for seedling stage samples. *PP2A*-*2* and *U6*-*1* were best for the plant stage, and *U6*-*1*, *EIF4A* and *PP2A*-*2* were the top three reference genes during the plant regeneration process in poplar. In addition, *PP2A*-*2* and *UBP* or *PP2A*-*2* and *bHLH* were the best combination as reference genes in the AR regeneration stage. This work will benefit future studies of expression and function analysis of miRNAs and their target genes in poplar.

## Methods

### Plant materials and tissue harvesting

The seedlings and plants of 84K poplar (*Populus alba* × *Populus glandulosa*) were grown in a tissue culture room under long-day conditions (16 h light/8 h dark) at 25/22 °C (day/night). Based on the Agrobacterium-mediated leaf disk transformation method [31, 32], the regeneration processes of poplar plants can be divided into three growth stages, including callus induction and shoots differentiation, seedlings on culture medium and plants in soil. Callus and shoot samples at different developmental stages were collected during shoot regeneration processes of 84K poplar [61], including callus induction stage at 4 days (Ca-4D), callus proliferation stage at 6 days (Ca-6D), callus expansion stage at 10 days (Ca-10D), callus transition stage at 12 days (Ca-12D), shoot emergence stage at 16 days (Shoot-16D) and the shoot elongation stage at 20 days (Shoot-20D). The samples from seedlings on the culture medium included the leaves (L-0.5M), stems (S-0.5M) and roots (R-0.5M) of 0.5-month-old seedlings as well as the leaves (L-1M), stems (S-1M) and roots (R-1M) of 1-month-old seedlings. The tissues from 3-month-old plants grown in soil included the shoot apical meristem (SAM-3M), unexpanded leaves (YL-3M), the first and second expanded leaves (ML-3M), the first to thirteenth internodes (N1-3M ~ N13-3M), the stem base (Base-3M), the roots (R-3M), and the root tips (RT-3M). The samples from the adventitious root (AR) developmental process included the AR induction stage at 18 h (AR-18H), the AR callus regeneration stage at 42 h and 60 h (AR-42H and AR-60H), the AR emergence stage at 4 and 5 days (AR-4D and AR-5D) and the AR elongation stage at 7 and 11 days (AR-7D and AR-11D) [61, 62]. Samples with three replicates were collected, immediately frozen and stored in liquid nitrogen.

### Total RNA extraction and cDNA synthesis

Total RNA was extracted using the LC sciences Total RNA Purification Kit (#TRK-1001, LC sciences, USA), which purifies all sizes of total RNA, including mRNA, ribosomal RNA, miRNA and other small RNA (20–200 nt), according to the previous methods with some modification [63]. The powder ground from 50 mg sample in liquid nitrogen was immediately transferred into a 2.0 ml RNase-free tube and added 600 μl extraction buffer with 6% Plant RNA Isolation Aid (Ambion, #Am9690). After shaking vigorously, the mixture was incubated in the ice for 15 min and then centrifuged at 12,000 rpm for 10 min at room temperate, by which the yield of total RNA could be improved. The remaining processes followed the manufacturer’s instructions. The integrity of total RNA was further assessed by 1.5% agarose gel electrophoresis, and the RNA concentration and purity were determined by NanoDrop™ 8000 Spectrophotometer (Thermo Fisher Scientific, USA). Only RNA samples with an A260/A280 ratio between 1.9 and 2.1 and A260/A230 greater than 1.80 were used for cDNA synthesis. Then, 1.5 μg of total RNA was polyadenylated with ATP by poly (A) polymerase (PAP) at 37 °C for 1 h in a 20-μl reaction mixture using the Poly(A) Tailing Kit (#AM1350, Invitrogen, USA). Then, 10 μl (750 ng) of the E-PAP-treated total RNA was reverse transcribed with a poly(T) adapter universal reverse transcription (RT)-primer (5′-AACGAGACGACGACAGACTTTTTTTTTTTTTTTV-3′) using SuperScript III reverse transcriptase Kit (#18080-051, Invitrogen, USA) following the manufacturer’s instruction. The cDNA was diluted 20-fold with nuclease-free water for qRT-PCR.

### Selection of candidate reference genes and primers design

In this study, twenty candidate genes were selected to identify the most stable reference gene(s) for quantification of miRNAs and mRNAs by qRT-PCR analysis. Base on a literature list of commonly used reference genes from ICG (http://icg.big.ac.cn/index.php/Main_Page) in 115 plants excluding polar [64], eight frequently used candidate reference genes, including Actin 7 (*ACT7*), Eukaryotic initiation factor 4A III (*EIF4A*), Glyceraldehyde-3-phosphate dehydrogenase (*GAPDH*), Histone (*Histone*), Protein phosphatase 2A-2 (*PP2A*-*2*), Protein phosphatase 2A subunit A2 (*PP2A*-*A2*), Ribosomal protein S18 (*RPS18*) and Polyubiquitin 10 (*UBQ10*) were selected. In addition, four genes with more stable expression levels in various vegetative and reproductive tissues at different developmental stages of *Populus tremula*, including ATP synthase subunit B (*ATPase*), Oligouridylate binding protein 1B (*UBP*), bHLH transcription factor (*bHLH*) and DNAJ homologue 2 (*DNAJ*), were selected for candidate reference genes according to the mean values and standard deviations of gene expression values (Additional file 3: Table S2) [60, 65]. Furthermore, three small noncoding RNAs (*U6*-*1*, *U6*-*2* and *5.8srRNA*) were also selected from GenBank, and three miRNAs (miR171, miR403 and miR482.1) were selected from miRBase (V21.0). The sequences of these candidate genes were cloned from the cDNA of 84K poplar and confirmed through sequencing. The primers (Table 1) were designed using oligo 7.0 software (Molecular Biology Insights, USA) based on the following criteria: primer lengths of 20-28 bp, GC contents of 45–55%, melting temperature (TM) of 60–63 °C and amplicon length of 100-250 bp.

### Quantitative real-time PCR analysis

Quantitative real-time PCR was conducted using LightCycler^®^ 96 Plates and performed on the LightCycler^®^ 480 System (Roche Molecular Systems, Germany). The reaction mixture contained 10 μl KAPA SYBR FAST qPCR Master Mix (# K4601, KAPA Biosystems, USA), 2 μl 20-fold diluted cDNA, 0.4 µM of each forward and reverse primer (Table 1) and ddH_2_O in a final volume of 20 μl. Amplifications were performed with the following program: 95 °C for 3 s; 40 cycles of 95 °C for 10 s, 60 °C for 30 s, 72 °C for 3 s; and melting curve analysis conditions (95 °C for 5 s, 65 °C increased to 95 °C with temperature increment of 0.11 °C every 1 s). No-template reactions were used as negative controls, and each sample was assessed in four technical replicates. Using a series of 10-fold diluted cDNA as templates, the standard curves were generated for each candidate reference gene. The correlation coefficient (R^2^) and slope were obtained from the linear regression model created by the LightCycle 480 system, and the PCR amplification efficiency (E) was calculated using the formulas E = 10^−1/*slope*^ − 1.

### Stability analysis for the candidate reference genes

To visualize the expression stability of the 18 candidate reference genes, the raw cycle threshold (Ct) values from different tissues and developmental stages were produced and calculated statistically by box plots and five different programs and algorithms, including geNorm [43], NormFinder [44], BestKeeper [45], the Delta CT method [66, 67] and the RankAggreg software [68]. The geNorm algorithm can calculate the average expression stability value (M), which is defined as the average pairwise variation in a particular gene with all other potential reference genes. The threshold of M value was set as 1.5, and genes with lowest M values exhibit the most stable expression. Additionally, geNorm also calculates the pairwise variation (V_n_ + V_n+1_) value that determines the optimal number of reference genes for accurate normalization with a cut-off value of V_n+1_ < 0.15 [43]. The NormFinder program uses an ANOVA-based model to consider intra- and inter-group variation in expression levels to calculate a stability value (SV) for expression, and a lower SV indicates increased stability [44]. Bestkeeper is an excel-based tool that determines the stability ranking of reference genes based on the coefficient of variance (CV) and the standard deviation (SD) of the average Ct values. The most stable gene exhibits the lowest CV ± SD value, and genes with SD higher than 1 were considered unacceptable and were excluded [45]. The Delta Ct method compares the relative expression of ‘pairs of genes’ within each sample. The stability of the reference gene is ranked according to a ‘process of elimination’ technique, by which genes can be compared against one another and either selected or eliminated on the basis of ΔCt among samples [66]. Finally, the raw Ct values of each gene were used to calculate the comprehensive ranking of reference genes using the RankAggreg software [68], which was based on the ranking of candidate references obtained from the four programs mentioned above. The program assigns an appropriate weight to an individual gene and calculates the geometric mean of the weight, providing an overall comprehensive ranking.

### Validation of identified reference genes

To examine the expression stability of potential reference genes, the relative expression levels of miR166 and its target gene *PtHB4* were analyzed in various tissues and AR developmental stages in poplar. The relative expression levels were normalized separately to the most stable and least stable reference genes analyzed by the four algorithms. The qRT-PCR amplification conditions of miRNA and genes were the same as described above. The relative expression levels of these genes were calculated according to the 2^−∆∆Ct^ method and presented as fold-change [69].

## Additional files

**Additional file 1: Fig. S1.** The melting curves of candidate reference genes. **Fig. S2.** The PCR amplification specificities of candidate reference genes detected by agarose gel electrophoresis.

**Additional file 2: Fig. S3.** The sequence similarity of candidate reference genes compared between 84K poplar and *Populus trichocarpa*.

**Additional file 3: Table S1.** The average of Ct values in different tissues during plant and AR regeneration of Poplar. **Table S2.** The normalized expression values of 14 candidate reference genes in various vegetative and reproductive tissues at different developmental stages of *Populus tremula*.
