# Supplementary material for: Selection and validation of reference genes for quantitative expression analysis of miRNAs and mRNAs in Poplar
Source: Plant Methods. 2019 Apr 6;15:35. doi: 10.1186/s13007-019-0420-1 (PMC6451301; doi:10.1186/s13007-019-0420-1)
Supplement: Supplementary file 1 — Additional file 1: Fig. S1. The melting curves of candidate reference genes. Fig. S2. The PCR amplification specificities of candidate reference genes detected by agarose gel electrophoresis. [file 13007_2019_420_MOESM1_ESM.pdf]

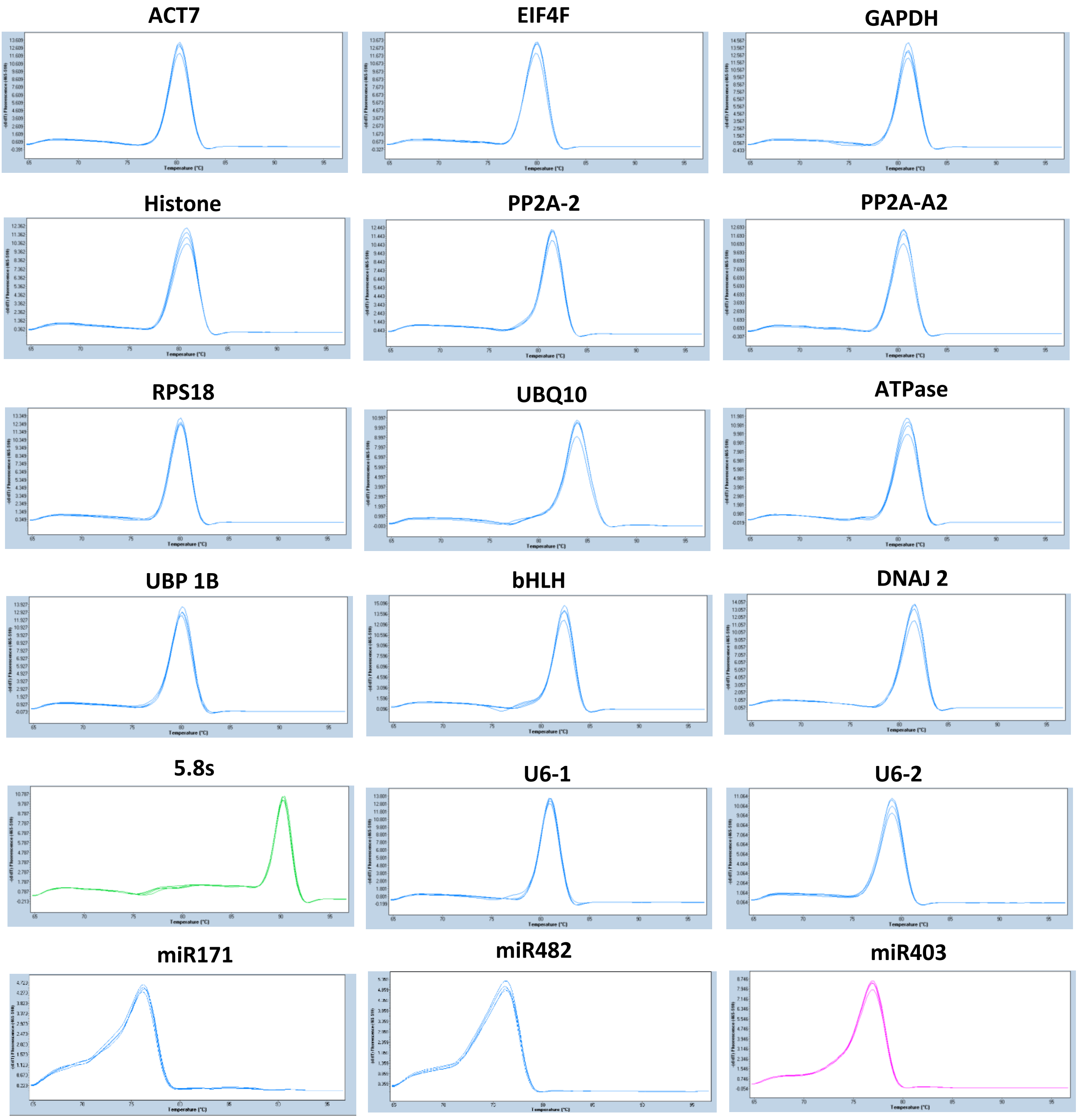

Figure S1. The melting curves of candidate reference genes.

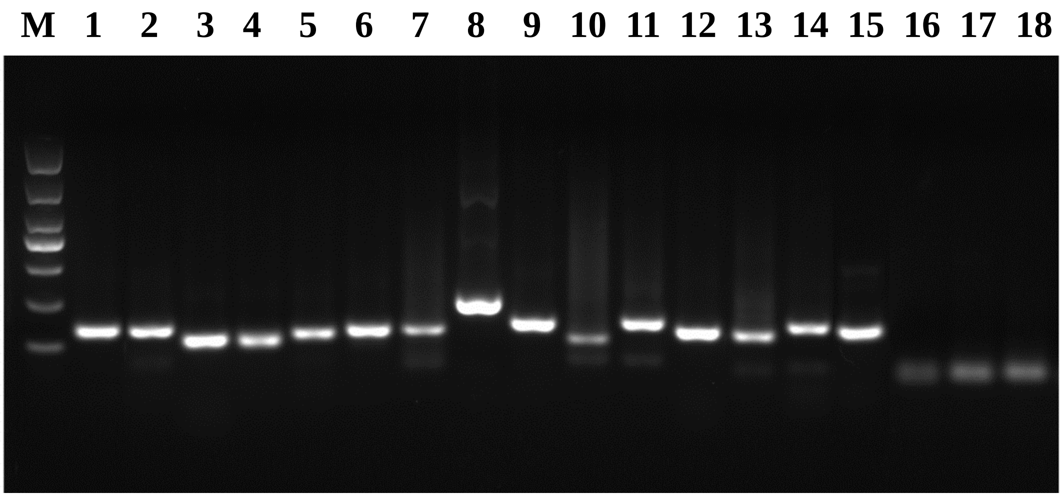

Figure S2. The PCR amplification specificities of candidate reference genes detected by agarose gel electrophoresis.

M, D2000 marker; 1, *ACT7*; 2, *EIF4A*; 3, *GAPDH*; 4, *Histone*; 5, *PP2A-2*; 6, *PP2A-A2*; 7, *RPS18*; 8, *UBQ10*; 9. *ATPase*; 10, *UBP*; 11, *bHLH*; 12, *DNAJ* ; 13, *U6-1*; 14, *U6-2*; 15, *5.8s*; 16, miR171; 17, miR482; 18, miR403.
