# Supplementary material for: Selection and validation of reference genes for quantitative expression analysis of miRNAs and mRNAs in Poplar
Source: Plant Methods. 2019 Apr 6;15:35. doi: 10.1186/s13007-019-0420-1 (PMC6451301; doi:10.1186/s13007-019-0420-1)
Supplement: Supplementary file 2 — Additional file 2: Fig. S3. The sequence similarity of candidate reference genes compared between 84K poplar and Populus trichocarpa. [file 13007_2019_420_MOESM2_ESM.pdf]

Figure S3: The sequence similarity of candidate reference genes compared between 84K poplar and *Populus trichocarpa*.

1. ACT7 Sequence similarity: 100%

```
J803231068_14-1.SEQ            GTGATCTCCTTGCTCATTCGGTCAGCAATACCAGGGAACATAGTGGAACCACCACTGAGC   60
J803231068_14-2.SEQRC          GTGATCTCCTTGCTCATTCGGTCAGCAATACCAGGGAACATAGTGGAACCACCACTGAGC   60
J803231068_14-3.SEQ            GTGATCTCCTTGCTCATTCGGTCAGCAATACCAGGGAACATAGTGGAACCACCACTGAGC   60
J803231068_14-4.SEQ            GTGATCTCCTTGCTCATTCGGTCAGCAATACCAGGGAACATAGTGGAACCACCACTGAGC   60
J803231068_14-5.SEQRC          GTGATCTCCTTGCTCATTCGGTCAGCAATACCAGGGAACATAGTGGAACCACCACTGAGC   60
POTRI.001G309500-ACT7.SEQRC    GTGATCTCCTTGCTCATTCGGTCAGCAATACCAGGGAACATAGTGGAACCACCACTGAGC   60
Consensus                      gtgatctccttgctcattcggtcagcaataccagggaacatagtggaaccaccactgagc

J803231068_14-1.SEQ            ACAATGTTACCATACAGATCCTTTCTAATATCCACATCACACTTCATGATTGAGTTGTAT  120
J803231068_14-2.SEQRC          ACAATGTTACCATACAGATCCTTTCTAATATCCACATCACACTTCATGATTGAGTTGTAT  120
J803231068_14-3.SEQ            ACAATGTTACCATACAGATCCTTTCTAATATCCACATCACACTTCATGATTGAGTTGTAT  120
J803231068_14-4.SEQ            ACAATGTTACCATACAGATCCTTTCTAATATCCACATCACACTTCATGATTGAGTTGTAT  120
J803231068_14-5.SEQRC          ACAATGTTACCATACAGATCCTTTCTAATATCCACATCACACTTCATGATTGAGTTGTAT  120
POTRI.001G309500-ACT7.SEQRC    ACAATGTTACCATACAGATCCTTTCTAATATCCACATCACACTTCATGATTGAGTTGTAT  120
Consensus                      acaatgttaccatacagatcctttctaatatccacatcacacttcatgattgagttgtat

J803231068_14-1.SEQ            GTAGTCTCGTGGATG                                               135
J803231068_14-2.SEQRC          GTAGTCTCGTGGATG                                               135
J803231068_14-3.SEQ            GTAGTCTCGTGGATG                                               135
J803231068_14-4.SEQ            GTAGTCTCGTGGATG                                               135
J803231068_14-5.SEQRC          GTAGTCTCGTGGATG                                               135
POTRI.001G309500-ACT7.SEQRC    GTAGTCTCGTGGATG                                               135
Consensus                      gtagtctcgtggatg
```

2. *EIF4A* Sequence similarity: 99.51%

```
J803231103_21-1.SEQ            TACATTCATCGAATTGGTCGTTCTGGTCGTTTTGGACGGAAGGGTGTTGCTATTAACTTC   60
J803231103_21-2.SEQRC          TACATTCATCGA.TTGGTCGTTCTGGTCGTTTTGGACGGAAGGGTGTTGCTATTAACTTC   59
J803231103_21-3.SEQRC          TACATTCATCGAATTGGTCGTTCTGGTCGTTTTGGACGGAAGGGTGTTGCTATTAACTTC   60
J803231103_21-4.SEQRC          TACATTCATCGAATTGGTCGTTCTGGTCGTTTTGGACGGAAGGGTGTTGCTATTAACTTC   60
J803231103_21-5.SEQ            TACATTCATCGAATTGGTCGTTCTGGTCGTTTTGGACGGAAGGGTGTTGCTATTAACTTC   60
POTRI.005G093900-EIF4A.SEQ     TACATTCATCGAATTGGTCGTTCTGGTCGTTTTGGACGGAAGGGTGTTGCTATTAACTTC   60
Consensus                      tacattcatcgaattggtcgttctggtcgttttggacggaagggtgttgctattaacttc

J803231103_21-1.SEQ            GTCAAAAGTGATGATATCAGGATTTTAAGAGATATTGAACAGTATTACAGCACCCAGATT  120
J803231103_21-2.SEQRC          GTCAAAAGTGATGATATCAGGATTTTAAGGGATATTGAACAGTATTACAGCACCCAGATT  119
J803231103_21-3.SEQRC          GTCAAAAGTGATGATATCAGGATTTTAAGAGATATTGAACAGTATTACAGCACCCAGATT  120
J803231103_21-4.SEQRC          GTCAAAAGTGATGATATCAGGATTTTAAGGGATATTGAACAGTATTACAGCACCCAGATT  120
J803231103_21-5.SEQ            GTCAAAAGTGATGATATCAGGATTTTAAGGGATATTGAACAGTATTACAGCACCCAGATT  120
POTRI.005G093900-EIF4A.SEQ     GTCAAAAGTGATGATATCAGGATTTTAAGAGATATTGAACAGTATTACAGCACCCAGATT  120
Consensus                      gtcaaaagtgatgatatcaggattttaagagatattgaacagtattacagcacccagatt

J803231103_21-1.SEQ            GACGAAATGCCTATGA                                              136
J803231103_21-2.SEQRC          GACGAAATGCCTATGA                                              135
J803231103_21-3.SEQRC          GACGAAATGCCTATGA                                              136
J803231103_21-4.SEQRC          GACGAAATGCCTATGA                                              136
J803231103_21-5.SEQ            GACGAAATGCCTATGA                                              136
POTRI.005G093900-EIF4A.SEQ     GACGAAATGCCTATGA                                              136
Consensus                      gacgaaatgcctatga
```

3. *GAPDH* Sequence similarity: 98.90%

```
J803231108_22-1.SEQ            AACCGACTTCATTGGTGACAACCGGTCAAGTATCTTTGATGCCAAAGCTGGAATTGCTTT   60
J803231108_22-2.SEQ            AACCGACTTCATTGGTGACAACCGGTCAAGTATCTTTGATGCCAAAGCTGGAATTGCTTT   60
J803231108_22-3.SEQRC          AACCGACTTCATTGGTGACAACCGATCAAGTATCTTTGATGCCAAAGCTGGAATTGCTCT   60
J803231108_22-4.SEQRC          AACCGACTTCATTGGTGACAACCGGTCAAGTATCTTTGATGCCAAAGCTGGAATTGCTTT   60
J803231108_22-5.SEQ            AACCGACTTCATTGGTGACAACCGATCAAGTATCTTTGATGCCAAAGCTGGAATTGCTCT   60
POTRI.012G094100-GAPDH.SEQ     AACCGACTTCATTGGTGACAACCGGTCAAGTATCTTTGATGCCAAAGCTGGAATTGCTTT   60
Consensus                      aaccgacttcattggtgacaaccggtcaagtatctttgatgccaaagctggaattgcttt

J803231108_22-1.SEQ            GAACGATAACTATGTGAAGCTCGTTGCGTGGTATGACAATGAGTG                105
J803231108_22-2.SEQ            GAACGATAACTATGTGAAGCTCGTTGCGTGGTATGACAATGAGTG                105
J803231108_22-3.SEQRC          GAACGAGAACTATGTGAAGCTCGTTGCGTGGTATGACAATGAGTG                105
J803231108_22-4.SEQRC          GAACGATAACTATGTGAAGCTCGTTGCGTGGTATGACAATGAGTG                105
J803231108_22-5.SEQ            GAACGAGAACTATGTGAAGCTCGTTGCGTGGTATGACAATGAGTG                105
POTRI.012G094100-GAPDH.SEQ     GAACGATAACTATGTGAAACTCGTTGCGTGGTATGACAATGAGTG                105
Consensus                      gaacgataactatgtgaagctcgttgcgtggtatgacaatgagtg
```

4. *Histone* Sequence similarity: 94.13%

```
J803231113_23-1.SEQ                  CTTAAAATCCTGGGCAATTTCACGAACAAGACGCTGGAAAGGTAACTTGCGGATCAAAAG   60
J803231113_23-2.SEQ                  CTTAAAATCCTGGGCAATTTCACGAACAAGACGCTGGAAAGGTAACTTGCGGATCAAAAG   60
J803231113_23-3.SEQRC                CTTAAAATCCTGGGCAATTTCACGAACAAGCCTCTGGAAAGGGAGTTTCCTGATCAGGAG   60
J803231113_23-4.SEQRC                CTTAAAATCCTGGGCAATTTCACGAACAAGACGCTGGAAAGGTAACTTGCGGATCAAAAG   60
J803231113_23-5.SEQRC                CTTAAAATCCTGGGCAATTTCACGAACAAGCCTCTGGAAAGGGAGTTTCCTGATCAGGAG   60
POTRI.005G072300-HISTONE.SEQRC       CTTAAAATCCTGGGCAATTTCACGAACAAGCCTCTGGAAAGGGAGTTTCCTAATCAGGAG   60
Consensus                            cttaaaatcctgggcaatttcacgaacaagacgctggaaagggaacttccggatcaaaag

J803231113_23-1.SEQ                  CTCAGTGCTCTTTTGGTACTTACGGATTTCACGAAGAGCAACAG                  104
J803231113_23-2.SEQ                  CTCAGTGCTCTTTTGGTACTTACGGATTTCACGAAGAGCAACAG                  104
J803231113_23-3.SEQRC                CTCAGTACTCTTCTGATACTTACGGATTTCACGAAGAGCAACAG                  104
J803231113_23-4.SEQRC                CTCAGTGCTCTTTTGGTACTTACGGATTTCACGAAGAGCAACAG                  104
J803231113_23-5.SEQRC                CTCAGTACTCTTCTGATACTTACGGATTTCACGAAGAGCAACAG                  104
POTRI.005G072300-HISTONE.SEQRC       CTCAGTACTCTTCTGATACTTACGGATTTCACGAAGAGCAACAG                  104
Consensus                            ctcagtactcttctgatacttacggatttcacgaagagcaacag
```

5. *PP2A-2* Sequence similarity: 99.30%

```
J803231004_1-2.SEQ                  TTTGGCGCACTGAACACTGTAACCACATTCTTGTCCTGGGACCAATTGTATCCTTCCATA   60
J803231005_1-3.SEQ                  TTTGGCGCACTGAACACTGTAACCACATTCTTGTCCTGGGACCAATTGTATCCTTCCATA   60
J803231006_1-4.SEQ                  TTTGGCGCACTGAACACTGTAACCACATTCTTGTCCTGGGACCAATTGTATCCTTCCATG   60
J803231007_1-5.SEQ                  TTTGGCGCACTGAACACTGTAACCACATTCTTGTCCTGGGACCAATTGTATCCTTCCATA   60
POTRI.015G068300-PP2A-2.SEQRC       TTTGGCGCACTGAACACTGTAACCACATTCTTGTCCTGGGACCAATTGTATCCTTCCATG   60
Consensus                           tttggcgcactgaacactgtaaccacattcttgtcctgggaccaattgtatccttccata

J803231004_1-2.SEQ                  ACAAGCTGGTGAGCTCTAGAAATGAGAGCGAGCCCATTAGTGTGGTTGAACTG         113
J803231005_1-3.SEQ                  ACAAGCTGGTGAGCTCTAGAAATGAGAGCGAGCCCATTAGTGTGGTTGAACTG         113
J803231006_1-4.SEQ                  ACAAGCTGGTGAGCTCTAGAAATGAGAGTGAGCCCATTAGTGTGGTTGAACTG         113
J803231007_1-5.SEQ                  ACAAGCTGGTGAGCTCTAGAAATGAGAGCGAGCCCATTAGTGTGGTTGAACTG         113
POTRI.015G068300-PP2A-2.SEQRC       ACAAGCTGGTGAGCTCTAGAAATGAGAGTGAGCCCATTAGTGTGGTTGAACTG         113
Consensus                           acaagctggtgagctctagaaatgagagcgagcccattagtgtggttgaactg
```

6. *PP2A-A2* Sequence similarity: 98.69%

```
J803231008_2-1.SEQ                   CAATGCCTATCCTCTGCAAGCTCCACAATTGCTGGCAATAGAGATTGGGAAAGCAAATCA   60
J803231009_2-2.SEQ                   CAATGCCTATCCTCTGCAAGCTCAACAATTGCTGGCAATAGTGATTGGGATAGTAGATCA   60
J803231010_2-3.SEQ                   CAATGCCTATCCTCTGCAAGCTCAACAATTGCTGGCAATAGTGATTGGGATAGTAGATCA   60
J803231011_2-4.SEQRC                 CAATGCCTATCCTCTGCAAGCTCAACAATTGCTGGCAATAGTGATTGGGATAGTAGATCA   60
J803231012_2-5.SEQRC                 CAATGCCTATCCTCTGCAAGCTCAACAATTGCTGGCAATAGTGATTGGGATAGTAGATCA   60
POTRI.010G127500-PP2A-A2.SEQRC       CAATGCCTATCCTCTGCAAGCTCAACAATTGCTGGCAATAGTGATTGGGATAGTAGATCA   60
Consensus                            caatgcctatcctctgcaagctcaacaattgctggcaatagtgattgggatagtagatca

J803231008_2-1.SEQ                   ATTCCAATCACCTGATTGACTTGATCAAGTTTGCTGATAATATTCAGTCGCACATCAGGA  120
J803231009_2-2.SEQ                   ATCCCAATAACCTGATTGACTTGATCAAGCTTGCTGATAATGTTCAGTCGCACATCAGGA  120
J803231010_2-3.SEQ                   ATCCCAATAACCTGATTGACTTGATCAAGCTTGCTGATAATGTTCAGTCGCACATCAGGA  120
J803231011_2-4.SEQRC                 ATCCCAATAACCTGATTGACTTGATCAAGCTTGCTGATAATGTTCAGTCGCACATCAGGA  120
J803231012_2-5.SEQRC                 ATCCCAATAACCTGATTGACTTGATCAAGCTTGCTGATAATGTTCAGTCGCACATCAGGA  120
POTRI.010G127500-PP2A-A2.SEQRC       ATCCCAATAACCTGATTGACTTGATCAAGCTTGCTGATGATGTTCAGTCGCACATCAGGA  120
Consensus                            atcccaataacctgattgacttgatcaagcttgctgataatgttcagtcgcacatcagga

J803231008_2-1.SEQ                   AATTCA                                                        126
J803231009_2-2.SEQ                   AATTCA                                                        126
J803231010_2-3.SEQ                   AATTCA                                                        126
J803231011_2-4.SEQRC                 AATTCA                                                        126
J803231012_2-5.SEQRC                 AATTCA                                                        126
POTRI.010G127500-PP2A-A2.SEQRC       AATTCA                                                        126
Consensus                            aattca
```

7. *RPS18* Sequence similarity: 99.74%

```
J803231013_3-1.SEQ             AGGCTCATCATCTTATCAAATCCCTCTCTACAAATGCACCTTCAGATAATTGGATTGGAG    60
J803231014_3-2.SEQ             AGGCTCATCATCTTATCAAATCCCTCTCTACAAATGCACCTTCAGATAATTGGATTGGAG    60
J803231015_3-3.SEQ             AGGCTCATCATCTTATCAAATCCCTCTCTACAAATGCACCTTCAGATAATTGGATTGGAG    60
J803231015_3-4.SEQ             AGGCTCATCATCTTATCAAATCCCTCTCTACAAATGCACCTTCAGATAATTGGATTGGAG    60
J803231015_3-5.SEQ             AGGCTCATCATCTTATCAAATCCCTCTCTACAAATGCACCTTCAGATAATTGGATTGGAG    60
POTRI.006G170500-RPS18.SEQ     AGGCTCATCATCTTATCAAATCCCTCTCTACAAATGCTCCTTCAGATAATTGGATTGGAG    60
Consensus                      aggctcatcatcttatcaaatccctctctacaaatgcaccttcagataattggattggag

J803231013_3-1.SEQ             ACAATCAAAGCTCCAATTCTTTTGAGTCTGCTGATGGCTTTGAGCAACGAATATTTGGTG   120
J803231014_3-2.SEQ             ACAATCAAAGCTCCAATTCTTTTGAGTCTGCTGATGGCTTTGAGCAACGAATATTTGGTG   120
J803231015_3-3.SEQ             ACAATCAAAGCTCCAATTCTTTTGAGTCTGCTGATGGCTTTGAGCAACGAATATTTGGTG   120
J803231015_3-4.SEQ             ACAATCAAAGCTCCAATTCTTTTGAGTCTGCTGATGGCTTTGAGCAACGAATATTTGGTG   120
J803231015_3-5.SEQ             ACAATCAAAGCTCCAATTCTTTTGAGTCTGCTGATGGCTTTGAGCAACGAATATT.GGTG   119
POTRI.006G170500-RPS18.SEQ     ACAATCAAAGCTCCAATTCTTTTGAGTCTGCTGATGGCTTTGAGCAACGAATATTTGGTG   120
Consensus                      acaatcaaagctccaattcttttgagtctgctgatggctttgagcaacgaatatttggtg

J803231013_3-1.SEQ             GCATTG                                                         126
J803231014_3-2.SEQ             GCATTG                                                         126
J803231015_3-3.SEQ             GCATTG                                                         126
J803231015_3-4.SEQ             GCATTG                                                         126
J803231015_3-5.SEQ             GCATTG                                                         125
POTRI.006G170500-RPS18.SEQ     GCATTG                                                         126
Consensus                      gcattg
```

8. *UBQ10* Sequence similarity:99.22%

```
J803231028_6-1.SEQ             GTTGATTTTTGCTGGGAAGCAGCTTGAAGATGGGAGAACTCTTGCTGACTACAACATTCA    60
J803231029_6-2_.SEQ            GTTGATTTTTGCTGGGAAGCAGCTTGAAGATGGGAGAACTCTTGCTGACTACAACATTCA    60
J803231030_6-3.SEQ             GTTGATTTTTGCTGGGAAGCAGCTTGAAGATGGGAGGACTCTTGCTGACTACAACATTCA    60
J803231031_6-4.SEQRC           GTTGATTTTTGCTGGGAAGCAGCTTGAAGATGGGAGGACTCTTGCTGACTACAACATTCA    60
J803231032_6-5.SEQRC           GTTGATTTTTGCTGGGAAGCAGCTTGAAGATGGGAGAACTCTTGCTGACTACAACATTCA    60
POTRI.001G418500-UBQ10.SEQ     GTTGATTTTTGCTGGGAAGCAGCTTGAAGATGGGAGGACTCTTGCTGACTACAACATTCA    60
Consensus                      gttgatttttgctgggaagcagcttgaagatgggagaactcttgctgactacaacattca

J803231028_6-1.SEQ             GAAGGAGTCAACCCTTCACTTGGTGCTGCGTCTCCGTGGAGGAATGCAGCTTTTTGTCAA   120
J803231029_6-2_.SEQ            GAAGGAGTCAACCCTTCACTTGGTGCTGCGTCTCCGTGGAGGAATGCAGATTTTTGTCAA   120
J803231030_6-3.SEQ             GAAGGAGTCAACCCTTCACCTGGTGCTGCGTCTCCGTGGAGGAATGCAGATTTTTGTCAA   120
J803231031_6-4.SEQRC           GAAGGAGTCAACCCTTCACCTGGTGCTGCGTCTCCGTGGAGGAATGCAGATTTTTGTCAA   120
J803231032_6-5.SEQRC           GAAGGAGTCAACCCTTCACTTGGTGCTGCGTCTCCGTGGAGGAATGCAGATTTTTGTCAA   120
POTRI.001G418500-UBQ10.SEQ     GAAGGAGTCAACCCTTCACTTGGTGCTGCGTCTCCGTGGAGGAATGCAGATTTTTGTCAA   120
Consensus                      gaaggagtcaacccttcacttggtgctgcgtctccgtggaggaatgcagatttttgtcaa

J803231028_6-1.SEQ             GACTTTGACCGGAAAGACCATCACCCTGGAGGTCGAGAGCTCAGACACCATTGACAACGT   180
J803231029_6-2_.SEQ            GACTTTGACCGGAAAGACCATCACCCTGGAGGTCGAGAGCTCAGACACCATTGACAACGT   180
J803231030_6-3.SEQ             GACTTTGACCGGAAAGACCATCACCCTGGAGGTCGAGAGCTCGGACACCATTGACAACGT   180
J803231031_6-4.SEQRC           GACTTTGACCGGAAAGACCATCACCCTGGAGGTCGAGAGCTCGGACACCATTGACAACGT   180
J803231032_6-5.SEQRC           GACTTTGACCGGAAAGACCATCACCCTGGAGGTCGAGAGCTCAGACACCATTGACAACGT   180
POTRI.001G418500-UBQ10.SEQ     GACTTTGACCGGAAAGACCATCACTCTGGAGGTCGAGAGCTCAGACACCATTGACAACGT   180
Consensus                      gactttgaccggaaagaccatcaccctggaggtcgagagctcagacaccattgacaacgt

J803231028_6-1.SEQ             GAAGGCCAAGAT                                                   192
J803231029_6-2_.SEQ            GAAGGCCAAGAT                                                   192
J803231030_6-3.SEQ             GAAGGCCAAGAT                                                   192
J803231031_6-4.SEQRC           GAAGGCCAAGAT                                                   192
J803231032_6-5.SEQRC           GAAGGCCAAGAT                                                   192
POTRI.001G418500-UBQ10.SEQ     GAAGGCCAAGAT                                                   192
Consensus                      gaaggccaagat
```

9. ATPase Sequence similarity: 97.99%

```
J803231073_15-1.SEQ            ACTCATCCCACCCCTGATCTTACGGGATACATCACAGAGGGACAGATTTATATTGACAGG    60
J803231073_15-2.SEQRC          ACTCATCCCACCCCTGATCTTACGGGATACATCACAGAGGGACAGATTTATATTGACAGG    60
J803231073_15-4.SEQ            ACTCATCCCACCCCTGATCTTACGGGATACATCACAGAGGGACAGATTTATATTGACAGG    60
J803231073_15-5.SEQRC          ACTCATCCCACCCCTGATCTTACGGGATACATCACAGAGGGACAGATTTATATTGACAGG    60
POTRI.004G177500-ATPASE.SEQ    ACTCATCCCACCCCTGATCTTACGGGATACATCACAGAGGGACAGATTTATGTTGACAGG    60
Consensus                      actcatcccacccctgatcttacgggatacatcacagagggacagatttatattgacagg

J803231073_15-1.SEQ            CAGCTACACAATAGACAGATTTATCCTCCAATCAATGTCCTCCCATCTCTGTCTCGTCTC    120
J803231073_15-2.SEQRC          CAGCTACACAATAGACAGATTTATCCTCCAATCAATGTCCTCCCATCTCTGTCTCGTCTC    120
J803231073_15-4.SEQ            CAGCTACAAAATAGGCAGATTTATCCTCCTATCAACGTCCTCCCATCTCTGTCTCGTCTC    120
J803231073_15-5.SEQRC          CAGCTACACAATAGACAGATTTATCCTCCAATCAATGTCCTCCCATCTCTGTCTCGTCTC    120
POTRI.004G177500-ATPASE.SEQ    CAGCTACAAAATAGGCAGATTTATCCTCCTATCAACGTCCTCCCATCTCTGTCTCGTCTC    120
Consensus                      cagctacacaatagacagatttatcctccaatcaatgtcctcccatctctgtctcgtctc

J803231073_15-1.SEQ            ATGAAGAGTGCC.ATTGG                                              137
J803231073_15-2.SEQRC          ATGAAGAGTGCCCATTGG                                              138
J803231073_15-4.SEQ            ATGAAGAGTGCC.ATTGG                                              137
J803231073_15-5.SEQRC          ATGAAGAGTGCC.ATTGG                                              137
POTRI.004G177500-ATPASE.SEQ    ATGAAGAGTGCC.ATTGG                                              137
Consensus                      atgaagagtgcc attgg
```

10. *UBP* Sequence similarity: 99.10%

```
J803231048_10-1.SEQ          GGCTTTGTTTCATTCCGTAATCAGCAGGAGGCACAAAGTGCTATTAATGACTTAACTGGT    60
J803231048_10-2.SEQ          GGCTTTGTTTCATTCCGTAATCAGCAGGAGGCGCAAAGTGCTATTAATGACTTAACTGGT    60
J803231048_10-3.SEQRC        GGCTTTGTTTCATTCCCTAATCAGCAGGAGGCACAAAGTGCTATTAATGACTTAACTGGT    60
J803231048_10-4.SEQRC        GGCTTTGTTTCATTCCGTAATCAGCAGGAGGCACAAAGTGCTATTAATGACTTAACTGGT    60
J803231048_10-5.SEQRC        GGCTTTGTTTCATTCCGTAATCAGCAGGAGGCACAAAGTGCTATTAATGACATAACTGGT    60
POTRI.006G279600-UBP.SEQ     GGCTTTGTTTCATTCCGTAATCAGCAGGAGGCACAAAGTGCTATTAATGACTTAACTGGT    60
Consensus                    ggctttgtttcattccgtaatcagcaggaggcacaaagtgctattaatgacttaactggt

J803231048_10-1.SEQ          AAATGGCTTGGAAACAGGCAGATAAGATGCAATTGGGCAACTAAAGGTGT              110
J803231048_10-2.SEQ          GAATGGCTCGGAAACAGGCAGATAAGATGCAATTGGGCAACTAAAGGTGT              110
J803231048_10-3.SEQRC        AAATGGCTTGGAAACAGGCAGATAAGATGCAATTGGGCAACTAAAGGTGT              110
J803231048_10-4.SEQRC        AAATGGCTTGGAAACAGGCAGATGAGATGCAATTGGGCAACTAAAGGTGT              110
J803231048_10-5.SEQRC        AAATGGCTTGGAAACAGGCAGATAAGATGCAATTGGGCAACTAAAGGTGT              110
POTRI.006G279600-UBP.SEQ     AAATGGCTTGGAAACAGGCAGATAAGATGCAATTGGGCAACTAAAGGTGT              110
Consensus                    aaatggcttggaaacaggcagataagatgcaattgggcaactaaaggtgt
```

11. *bHLH* Sequence similarity: 97.73%

```
J803231078_16-1.SEQ           ATCTGAATCGTGTAGTGCGTCTAGCTCCAAAGCATGCAGGGAAAAGTTGCGAAGGGATAG    60
J803231078_16-2.SEQRC         ATCTGAATCGTGTAGTGCGTCTAGCTCCAAAGCATGTAGGGAAAAGCTGCGAGGGGATAG    60
J803231078_16-3.SEQRC         ATCTGAATCGTGTAGTGCGTCTAGCTCCAAAGCATGTAGGGAAAAGCTGCGAAGGGATAG    60
J803231078_16-4.SEQRC         ATCTGAATCGTGTAGTGCGTCTAGCTCCAAAGCATGCAGGGAAAAGTTGCGAAGGGATAG    60
J803231078_16-5.SEQRC         ATCTGAATCGTGTAGTGCGTCTAGCTCCAAAGCATGTAGGGAAAAGCTGCGAAGGGATAG    60
POTRI.011G132400-bHLH.SEQ     ATCTGAATCGTGTAGTGCGTCTAGCTCCAAAGCATGTAGGGAAAAGTTGCGAAGGGATAG    60
Consensus                     atctgaatcgtgtagtgcgtctagctccaaagcatgtagggaaaagctgcgaagggatag

J803231078_16-1.SEQ           GCTAAATGACAAGTTTATGGAATTGGGCTCTATCTTGGATCCTGGGAGAGCTCCCAAAAC    120
J803231078_16-2.SEQRC         GCTGAATGACAAGTTTATAGAATTGGGCTCTATCTTGGAGCCTGGAAGAACTCCCAAAAC    120
J803231078_16-3.SEQRC         GCTGAATGACAAGTTTATAGAATTGGGCTCTATCTTGGAGCCTGGAAGAACTCCCAAAAC    120
J803231078_16-4.SEQRC         GCTAAATGACAAGTTTATGGAATTGGGCTCTATCTTGGATCCTGGGAGAGCTCCCAAAAC    120
J803231078_16-5.SEQRC         GCTGAATGACAAGTTTATAGAATTGGGCTCTATCTTGGAGCCTGGAAGAACTCCCAAAAC    120
POTRI.011G132400-bHLH.SEQ     GCTAAATGACAAGTTTATGGAATTGGGCTCTATCTTGGATCCTGGGAGAACTCCCAAAAC    120
Consensus                     gctaaatgacaagtttatagaattgggctctatcttggagcctggaagaactcccaaaac

J803231078_16-1.SEQ           GGACAAGGCTGCTATTTTGGTTGATG                                      146
J803231078_16-2.SEQRC         GGACAAGGCTGCTATTTTGGTTGATG                                      146
J803231078_16-3.SEQRC         GGACAAGGCTGCTATTTTGGTTGATG                                      146
J803231078_16-4.SEQRC         GGACAAGGCTGCTATTTTGGTTGATG                                      146
J803231078_16-5.SEQRC         GGACAAGGCTGCTATTTTGGTTGATG                                      146
POTRI.011G132400-bHLH.SEQ     GGACAAGGCTGCTATTTTGGTTGATG                                      146
Consensus                     ggacaaggctgctattttggttgatg
```

12. *DNAJ 2* Sequence similarity: 99.78%

```
J803231003_1-1.SEQ            AGGCAATTAATGACAAGGACCGTTGCCCTCAATGCAAGGGTGAGAAGGTGGTCCAAGAGA    60
J803231088_18-1.SEQ           AGGCAATTAATGACAAGGACCGTTGCCCTCAATGCAAGGGTGAGAAGGTGGTCCAAGAGA    60
J803231088_18-2.SEQ           AGGCAATTAATGACAAGGACCGTTGCCCTCAATGCAAGGGTGAGAAGGTGGTCCAAGAGA    60
J803231088_18-3.SEQ           AGGCAATTAATGACAAGGACCGTTGCCCTCAATGCAAGGGTGAGAAGGTGGTCCAAGAGA    60
J803231088_18-4.SEQRC         AGGCAATTAATGACAAGGACCGTTGCCCTCAATGCAAGGGTGAGAAGGTGGTCCAAGAGA    60
J803231088_18-5.SEQ           AGGCAATTAATGACAAGGACCGTTGCCCTCAATGCAAGGGTGAGAAGGTGGTCCAAGAGA    60
POTRI.010G243100-DNAJ.SEQ     AGGCAATTAATGACAAGGACCGTTGCCCTCAATGCAAGGGTGAGAAGGTGGTCCAAGAGA    60
Consensus                     aggcaattaatgacaaggaccgttgccctcaatgcaagggtgagaaggtggtccaagaga

J803231003_1-1.SEQ            AAAAAGTTTTGGAAGTAGTTGTTGAGAAGGGCATGCAAAATGCACCGAGGATTACTTTCC   120
J803231088_18-1.SEQ           AAAAAGTTTTGGAAGTAGTTGTTGAGAAGGGCATGCAAAATTCACAGAGGATTACTTTCC   120
J803231088_18-2.SEQ           AAAAAGTTTTGGAAGTAGTTGTTGAGAAGGGCATGCAAAATGCACAGAGGATTACTTTCC   120
J803231088_18-3.SEQ           AAAAAGTTTTGGAAGTAGTTGTTGAGAAGGGCATGCAAAATGCACAGAGGATTACTTTCC   120
J803231088_18-4.SEQRC         AAAAAGTTTTGGAAGTAGTTGTTGAGAAGGGCATGCAAAATGCACAGAGGATTACTTTCC   120
J803231088_18-5.SEQ           AAAAAGTTTTGGAAGTAGTTGTTGAGAAGGGCATGCAAAATGCACAGAGGATTACTTTCC   120
POTRI.010G243100-DNAJ.SEQ     AAAAAGTTTTGGAAGTAGTTGTTGAGAAGGGCATGCAAAATGCACAGAGGATTACTTTCC   120
Consensus                     aaaaagttttggaagtagttgttgagaagggcatgcaaaatgcacagaggattactttcc

J803231003_1-1.SEQ            CTGGAGAGGC                                                     130
J803231088_18-1.SEQ           CTGGAGAGGC                                                     130
J803231088_18-2.SEQ           CTGGAGAGGC                                                     130
J803231088_18-3.SEQ           CTGGAGAGGC                                                     130
J803231088_18-4.SEQRC         CTGGAGAGGC                                                     130
J803231088_18-5.SEQ           CTGGAGAGGC                                                     130
POTRI.010G243100-DNAJ.SEQ     CTGGAGAGGC                                                     130
Consensus                     ctggagaggc
```

13. *U6-1* Sequence similarity: 98.54%

```
J803231018_4-2.SEQRC          GTGACCTTTATTGCGACATCCACTTGGGTCTATATGTAATTCGTGGCGAGAATGTTGTCT    60
J803231018_4-3.SEQRC          GTGACCTTTATTGCGACATCCACTTGGGTCTATATGTAATTCGTGGCGAGAATGTTGTCT    60
J803231018_4-4.SEQ            GTGACCTTTATTGCGACATCCACTTGGGTCTATATGTAATTCGTGGCGAGAATGTTGTCT    60
J803231018_4-5.SEQ            GTGACCTTTATTGCGACATCCACTTGGGTCTATATGTGATTCGTGGGGAGAATGTTGTCT    60
POTRI.001G166600-U6-1.SEQ     GTGACCTTTATTGCGACATCCACTTGGGTCTATATGTGATTCGTGGGGAGAATGTTGTCT    60
Consensus                     gtgacctttattgcgacatccacttgggtctatatgtaattcgtggcgagaatgttgtct

J803231018_4-2.SEQRC          TAATTGGGGAGCTGGATTTGGAGAGGGAGGAACTTCCACCACATATGACTCGTGTTTCAG   120
J803231018_4-3.SEQRC          TAATTGGGGAGCTGGATTTGGAGAGGGAGGAACTTCCACCACATATGACTCGTGTTTCAG   120
J803231018_4-4.SEQ            TAATTGGGGAGCTGGATTTGGAGAGGGAGGAACTTCCACCACATATGACTCGTGTTTCAG   120
J803231018_4-5.SEQ            TAATTGGAGAGCTGGATTTGGAGAGGGAGGAGCTTCCACCACATATGACTCGTGTT.CAG   119
POTRI.001G166600-U6-1.SEQ     TAATTGGAGAGCTGGATTTGGAGAGGGAGGAGCTTCCACCACATATGACTCGTGTTTCAG   120
Consensus                     taattggggagctggatttggagagggaggaacttccaccacatatgactcgtgtttcag

J803231018_4-2.SEQRC          AA                                                             122
J803231018_4-3.SEQRC          AA                                                             122
J803231018_4-4.SEQ            AA                                                             122
J803231018_4-5.SEQ            AA                                                             121
POTRI.001G166600-U6-1.SEQ     AA                                                             122
Consensus                     aa
```

14. *U6-2* Sequence similarity: 99.22%

```
J803231023_5-1.SEQ            TTCCGCGGATGAAAGCATCACCATATTTGTTTTTCAGTTGGCCATTTACATATTCCTCTG    60
J803231024_5-2.SEQ            TTCCTCGTATGAAAGCATCACCATATTTGTTTTTCAGTTGGCCATTTACATATTCTTCTG    60
J803231025_5-3.SEQRC          TTCCTCGTATGAAAGCATCACCATATTTGTTTTTCAGTTGGCCATTTACGTATTCCTCTG    60
J803231026_5-4.SEQRC          TTCCTCGTATGAAAGCATCACCATATTTGTTTTTCAGTTGGCCATTTACATATTCTTCTG    60
J803231026_5-5.SEQ            TTCCTCGTATGAAAGCATCACCATATTTGTTTTTCAGTTGGCCATTTACATATTCTTCTG    60
POTRI.008G078400-U6-2.SEQRC   TTCCTCGTATGAAAGCATCACCATATTTGTTTTTCAGTTGGCCATTTACATATTCTTCTG    60
Consensus                     ttcctcgtatgaaagcatcaccatatttgtttttcagttggccatttacatattcttctg

J803231023_5-1.SEQ            TTTGTTCCATTGCTATGTTCATGTACCCATCAAGACAAGCTAAAATACCTCTATAATCAA   120
J803231024_5-2.SEQ            TTTGTTCCATTGCTATGTTCATGTACCCATCAAGACAAGCTAAAATACCTCTATAATCAA   120
J803231025_5-3.SEQRC          TTTGTTCCATTGCTATGTTCATGTACCCATCAAGACAAGCTAAAATACCTCTATAATCAA   120
J803231026_5-4.SEQRC          TTTGTTCCATTGCTATGTTCATGTACCCATCAAGACAAGCTAAAATACCTCTATAATCAA   120
J803231026_5-5.SEQ            TTTGTTCCATTGCTATGTTCATGTACCCATCAAGACAAGCTAAAATACCTCTATAATCAA   120
POTRI.008G078400-U6-2.SEQRC   TTTGTTCCATTGCTATGTTCATGTACCCATCAAGACAAGCTAAAATACCTCTATAATCAA   120
Consensus                     tttgttccattgctatgttcatgtacccatcaagacaagctaaaatacctctataatcaa

J803231023_5-1.SEQ            CTCCTAAATTGAGCTTAACCACAACAGG                                   148
J803231024_5-2.SEQ            CTCCAGAATTGAGCTTAACCACAACAGG                                   148
J803231025_5-3.SEQRC          CTCCAGAATTGAGCTTAACCACAACAGG                                   148
J803231026_5-4.SEQRC          CTCCAGAATTGAGCTTAACCACAACAGG                                   148
J803231026_5-5.SEQ            CTCCAGAATTGAGCTTAACCACAACAGG                                   148
POTRI.008G078400-U6-2.SEQRC   CTCCAGAATTGAGCTTAACCACAACAGG                                   148
Consensus                     ctccagaattgagcttaaccacaacagg
```

15. *5.8S* Sequence similarity: 99.66%

```
5.8S-1.SEQ     ACGTCTGCCTGGGTGTCACGCATCGTCGCCCCCTCTCCCCTCGGCTCGCGAGGGCGGGGG    60
5.8S-2.SEQ     ACGTCTGCCTGGGTGTCACGCATCGTCGCCCCCTCTCCCCTCGACTCGCGAGGGCGGGGG    60
5.8S-3.SEQ     ACGTCTGCCTGGGTGTCACGCATCGTCGCCCCCTCTCCCCTCGACTCGCGAGGGCGGGGG    60
5.8S-4.SEQ     ACGTCTGCCTGGGTGTCACGCATCGTCGCCCCCTCTCCCCTCGGCTCGCGAGGGCGGGGG    60
5.8S-5.SEQ     ACGTCTGCCTGGGTGTCACGCATCGTCGCCCCCTCTCCCCTCGACTCGCGAGGGCGGGGG    60
5.8S.SEQ       ACGTCTGCCTGGGTGTCACGCATCGTCGCCCCCTCTCCCCTCGGCTCGCGAGGGCGGGGG    60
Consensus      acgtctgcctgggtgtcacgcatcgtcgcccccctctcccctcgactcgcgagggcggggg

5.8S-1.SEQ     CGGATACTGGTCTCCCGCGCGCTCCCGCTCGCGGTTGGCCCAAAATCGAGTCCTCGGCGA   120
5.8S-2.SEQ     CGGATACTGGTCTCCCGCGCGCTCCCGCTCGCGGTTGGCCCAAAATCGAGTCCTCGGCGA   120
5.8S-3.SEQ     CGGATACTGGTCTCCCGCGCGCTCCCGCTCGCGGTTGGCCCAAAATCGAGTCCTCGGCGA   120
5.8S-4.SEQ     CGGATACTGGTCTCCCGCGCGCTCCCGCTCGCGGTTGGCCCAAAATCGAGTCCTCGGCGA   120
5.8S-5.SEQ     CGGATACTGGTCTCCCGCGCGCTCCCGCTCGCGGTTGGCCCAAAATCGAGTCCTCGGCGA   120
5.8S.SEQ       CGGATACTGGTCTCCCGCGCGCTCCCGCTCGCGGTTGGCCCAAAATCGAGTCCTCGGCGA   120
Consensus      cggatactggtctcccgcgcgctcccgctcgcggttggcccaaaatcgagtcctcggcga

5.8S-1.SEQ     CGGTCGCCACGACGAGCGGTGGTTG                                      145
5.8S-2.SEQ     CGGTCGCCACGACGAGCGGTGGTTG                                      145
5.8S-3.SEQ     CGGTCGCCACGACGAGCGGTGGTTG                                      145
5.8S-4.SEQ     CGGTCGCCACGACGAGCGGTGGTTG                                      145
5.8S-5.SEQ     CGGTCGCCACGACGAGCGGTGGTTG                                      145
5.8S.SEQ       CGGTCGCCACGACGAGCGGTGGTTG                                      145
Consensus      cggtcgccacgacgagcggtggttg
```

16. miR171 Sequence similarity: 98.33%

```
miR171-1.SEQ        AGATTGAGCCGCGCCAATATCAAAAAAAAAAAGTCTGTCGTCGTCTCGT    49
miR171-2.SEQ        AGATTGAGCCGCGCCAATATCAAAAAAAAAAAGTCTGTCGTCGTCTCGT    49
miR171-3.SEQ        AGATTGAGCCGCGCCAATATCAAAAAAAAAG..TCTGTCGTCGTCTCGT    47
miR171-4.SEQ        AGATTGAGCCGCGCCAATATCAAAAAAAAAAAGTCTGTCGTCGTCTCGT    49
miR171-5.SEQ        AGATTGAGCCGCGCCAATATCAAAAAAAAAA.GTCTGTCGTCGTCTCGT    48
ptc-miR171c.SEQ     AGATTGAGCCGCGCCAATATCAAAAAAAAAA.GTCTGTCGTCGTCTCGT    48
Consensus           agattgagccgcgccaatatcaaaaaaaaaaagtctgtcgtcgtctcgt
```

17. miR403 Sequence similarity: 98.62%

```
J803231058_12-2.SEQ       CGCGTTAGATTCACGCACAAACTCGAAAAAAAAAAAAA..GTCTGTCGTCGTCTCGT    55
J803231058_12-3.SEQRC     CGCGTTAGATTCACGCACAAACTCGAAAAAAAAAAAAAAAGTCTGTCGTCGTCTCGT    57
J803231058_12-4.SEQRC     CGCGTTAGATTCACGCACAAACTCAAAAAAAAAAAAAAAAGTCTGTCGTCGTCTCGT    57
J803231058_12-5.SEQ       CGCGTTAGATTCACGCACAAACTCGAAAAAAAAAAAAAA.GTCTGTCGTCGTCTCGT    56
Ptc-miR403.SEQ            CGCGTTAGATTCACGCACAAACTCGAAAAAAAAAAAAAAAGTCTGTCGTCGTCTCGT    57
Consensus                 cgcgttagattcacgcacaaactcgaaaaaaaaaaaaaaagtctgtcgtcgtctcgt
```

18. miR482 Sequence similarity: 98.69%

```
MIR482-1.SEQ       CCTACTCCTCCCATTCCAAAAAAAAAAAAAAAAAGTCTGTCGTCGTCTCGT    50
MIR482-2.SEQ       CCTACTCCTCCCATTCCAAAAAAAAAAAAAAAAAGTCTGTCGTCGTCTCGT    50
MIR482-3.SEQ       CCTACTCCTCCCATTCCAAAAAAAAAAAAAAAA.GTCTGTCGTCGTCTCGT    49
MIR482-4.SEQ       CCTACTCCTCCCATTCCAAAAAAAAAAAAAAAA.GTCTGTCGTCGTCTCGT    49
MIR482-5.SEQ       CCTACTCCTCCCATTCCAAAAAAAAAAAAAAAA.GTCTGTCGTCGTCTCGT    49
ptc-miR482.SEQ     CCTACTCCTCCCATTCCAAAAAAAAAAAAAAAA.GTCTGTCGTCGTCTCGT    49
Consensus          cctactcctcccattccaaaaaaaaaaaaaaaa gtctgtcgtcgtctcgt
```
